# Efficient Adsorptive Removal of Methyl Violet Dye from Wastewater Using Functionalized Groundnut Shell Biochar: Application, Mechanism, Modeling, and Optimization Study

**DOI:** 10.1002/gch2.70161

**Published:** 2026-09-28

**Authors:** Md. Asik Uzzaman Asa, Md. Mahmudur Rahman, Jubayer Ahmed, Bijoy Chandra Ghos, Salah Knani, Mustafizur Rahman Naim, Reem Alreshidi, Md. Ismail Hossain

**Affiliations:** ^1^ Department of Chemical Engineering Rajshahi University of Engineering and Technology (RUET) Rajshahi Bangladesh; ^2^ BCSIR Rajshahi Laboratory Bangladesh Council of Scientific and Industrial Research (BCSIR) Rajshahi Bangladesh; ^3^ Center for Scientific Research and Entrepreneurship Northern Border University Arar Saudi Arabia; ^4^ Biomedical and Toxicological Research Institute (BTRI) Bangladesh Council of Scientific and Industrial Research (BCSIR) Dhaka Bangladesh; ^5^ Department of Physics College of Science Northern Border University Arar Saudi Arabia

**Keywords:** adsorption of Methyl violet (MV) dye, box‐behnken design and response surface methodology, functionalized ground nutshell biochar (FGNB), industrial wastewater management, residual mass of groundnut shell after MeOH extraction, synergistic adsorption mechanism and mathematical modeling

## Abstract

This investigation focuses on the abatement of methyl violet (MV) dye from industrial effluents using the deployment of functionalized biochar prepared from methanol‐extracted groundnut shell residue. The biochar was functionalized by 30% tartaric acid(C_4_H_4_O_6_) to boost the surface functionality and adsorption performance. It was determined by conducting SEM, FTIR, XRD, and ZP analysis that a porous structure was developed with high concentrations of oxygen‐carrying functional groups and favorable surface charge qualities. Batch adsorption studies were designed to evaluate the effect of influential parameters on the removal performance. The equilibrium behavior was successfully interpreted using several mathematical models. The maximum adsorption capacity was found ∼88.57 mg/g. The kinetic and thermodynamic evaluation revealed that both pseudo‐first‐order and pseudo‐second‐order models closely represent the experimental data, suggesting that the adsorption behavior arises from multiple interacting synergistic pathways rather than a single dominant mechanism, and the adsorption proceeds spontaneously, followed by an endothermic process. Additionally, the Box‐Behnken and experimental designs were used to show that the system is highly sensitive to the relationship between operational parameters. The results, when combined, reveal that the FGNB biochar derived from secondary waste biomass provides a possible solution to dye‐contaminated wastewater treatment that is economically feasible and eco‐friendly.

## Graphical abstract

This study focused on the production and application of secondary waste, namely ground nutshell residual mass (after MeOH extraction)‐derived FGNB‐based multifunctional nanoadsorbents in industrial wastewater treatment technology. The FGNB nanosorbent would be a sustainable alternative to fossil‐based, hazardous ones for environmental protection and would facilitate a zero‐waste strategy. The synergistic mechanism and modeling could be beneficial for improved prediction and data interpretation.

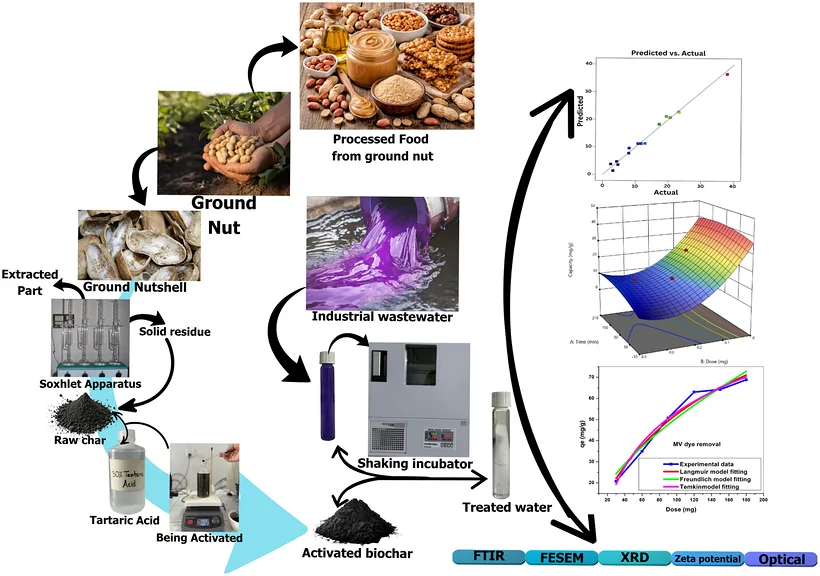

**Abbreviations**
FGNB: Functionalized Ground Nutshell Biochar
BCSIR: Bangladesh Council of Scientific and Industrial Research
CrI: Crystalline Index
DI Water: Deionized Water
BBD: Box–Behnken Design
FESEM: Field Emission Scanning Electron Microscope
FTIR‐ATR: Fourier Transform Infrared Spectroscopy—Attenuated Total Reflectance
FWHM: Full Width at Half Maximum
MV: Methyl Violet
RSM: Response Surface Methodology
UV–Vis–NIR: UV–vis‐Near Infrared Spectroscopy
RSM: Response Surface Methodology
ANOVA: Analysis of Variance
XRD: X‐ray Diffraction

## 1 Introduction

Rapid industrialization and urbanization have significantly increased the generation of industrial wastewater, posing serious challenges to global water sustainability. Industries such as textile, leather, paint, printing, cosmetics, and metal processing discharge large volumes of dye‐containing effluents, many of which are released into natural water bodies without adequate treatment, particularly in developing countries [1] A major environmental concern arises from the widespread practice in many developing countries, including Bangladesh, where industrial effluents are often discharged directly into natural water bodies without adequate treatment. These discharges contaminate both groundwater and surface water systems, such as rivers, canals, lakes, ponds, and coastal regions. Reports from the Bangladesh Department of Environment indicate that tannery industries alone release approximately 21,600 m^3^ (5.7 million gallons) of hazardous waste into rivers daily. In addition, the Dhaka Water Supply and Sewerage Authority (WASA) has reported that nearly 12,000 m^3^ (420,000 cubic feet) of untreated wastewater is continuously discharged into water bodies surrounding industrial zones such as Tejgaon, Badda, and Mohakhali, with textile washing and dyeing operations identified as the primary contributors [2]. It was estimated that one textile industry alone generated a waste and sludge of up to 56 million tons annually and 0.5 million tons yearly [3]. Synthetic dyes and toxic pollutants that are contained in these wastewaters cause serious threats to human health and aquatic life. Over 10,000 unique dye compounds have been produced in the world, with an aggregate production of over 700 thousand tons per annum. These dyes find a lot of application in those industries, mainly in adding color to a variety of products [4, 5]. MV is a commonly used dye in these industrial sectors, which has poor fixation and hydrolyzes, leading to the release of high proportions into water bodies. Its abundance reduces penetration of light, obscures photosynthesis, interferes with metabolism in the water, leading to ecotoxicity [6, 7]. Moreover, synthetic dyes like MV are one of the most toxic xenobiotics [8, 9]; these compounds, once introduced into the human body, are likely to remain within the vital organs, and they never get removed [10, 11]. This carcinogenic dye is associated with severe chronic disorders, including neurotoxicity, cardiovascular complications, cytotoxicity, carcinogenicity, hepatotoxicity, nephrotoxicity, endocrine disruption, inhibition of hemoglobin synthesis, paralysis, and, in extreme cases, mortality [12]. Moreover, such environmental contamination directly conflicts with the United Nations Sustainable Development Goal 6 (SDG 6) [13], which emphasizes the provision of clean water and adequate sanitation. The continued discharge of untreated pollutants contributes to the deterioration of safe drinking water sources and exacerbates the lack of proper sanitation facilities for the general population [14, 15]. So, it is significant that these dyes be eliminated in effluents before the effluents are released into water bodies as a measure of protection to the health of people and the ecosystems as well [16]. However, several wastewater treatment methods do exist like Physical treatment methods; including sedimentation and filtration, membrane filtration [17, 18, 19], coagulation–flocculation [20] electrocoagulation/electro flotation [21, 22], Chemical treatment methods [23], Biological treatment methods, Photocatalysis, Adsorption (e.g., activated carbon, biochar, nanomaterials) etc. which are used to purify real‐time effluence like MV dye; disintegrated by those industries [24]. Among the available wastewater treatment technologies, adsorption has emerged as a promising approach because of its cost‐effectiveness, simple operation, low energy consumption, and high pollutant removal efficiency [25]. In contrast, many conventional treatment methods suffer from inherent drawbacks [26, 27], including elevated operational and disposal costs, potential formation of toxic byproducts [28], complex process optimization, high labor and energy demands, technological limitations, and the possibility of generating secondary pollutants [29, 30, 31]. This study has, therefore, followed the batch adsorption methodology to treat the huge industrial effluent for its ingenuity and low expenditure, fast processing and high removal efficiency, flexibility in operations, accurate kinetics, and isotherm studies [32, 33]. Although activated carbon commercially obtained is widely used in the batch process as an adsorbent, it is quite expensive; this limits its use on a large scale [34]. Consequently, more interest has been focused on the design of alternative adsorbents that are cheap, sustainable, and made of waste products. The main goal of such processes is to attain effective dye elimination in wastewater and minimize total treatment costs. Agro‐industrial residues represent an abundant and underutilized biomass resource that can be converted into efficient adsorbents for wastewater treatment. These resources may be transformed into useful bio‐adsorbents having significant adsorption capacity. These materials have also been successfully used as dye removers in several studies with rice husk‐derived charcoal [35], mahogany bark charcoal [36], and jute stick‐based charcoal [37] among others. As an abundant agro‐industrial byproduct, groundnut shells are easily accessible during peanut processing and are generally a noxious byproduct or waste that is usually dumped or burned, causing environmental disposal problems [38]. This ground nutshell can be separated into two parts after extraction: one is the extracted part, and the other is the residual part. After extraction of the extracted part, which is generally an active material, it is widely accepted for pharmaceutical or biomedical uses. However, approximate 1% of the ground nutshell's total mass is really extracted; the rest (more than 99%) is regarded as waste, which is enormous on an industrial scale and has no discernible purpose. This discarded fraction, referred to as residual mass, primarily constitutes solid waste and poses significant health risks. Improper disposal of these residues may contribute to air pollution and has been associated with respiratory illnesses, including asthma and (COPD) pulmonary infections, and, in severe cases, lung cancer [39]. In addition to being detrimental to the economy, this huge number of residual parts disrupts biodiversity and, most significantly, produces secondary pollutants. In this work, using this waste in line, it is possible to create something valuable from this waste, which is consistent with the circular economy [40]. Hence, the proposed approach demonstrates the potential of converting methanol‐extracted groundnut shell residue into a value‐added adsorbent, thereby contributing to biomass waste valorization and sustainable wastewater treatment. Moreover, the material has a high content of lignocellulose, which adds to the occurrence of several surface functional groups, engaging hydroxyl (─OH), carbonyl (─C═O), and carboxyl (─COOH), which are important as active binding sites in the course of the biosorption process [41]. Additionally, it serves as a sustainable bio‐adsorbent that demonstrated high dye removal efficiency in the present study [42, 43]. Conversely, the methanol‐extracted residual groundnut shell, which retains abundant cellulose and other functional biopolymers, can be valorized as a promising precursor for the production of high‐performance adsorbents. This potential arises from its favorable physicochemical characteristics, robust thermomechanical stability, well‐defined morphology, and environmentally sustainable nature [44]. The valorization of agro‐industrial residues as adsorbents provides a practical and sustainable alternative to commercial adsorbents, reducing material costs while supporting wastewater treatment in resource‐limited regions [34]. Hence, the residual mass of groundnut shells can be an outstanding adsorbent that should be utilized in developing countries such as Bangladesh as a cheaper substitute. The adsorption systems are affected by many parameters during operation, which include the pH of the solution, the quantity of pollutant present in it, the dosage of adsorbent used, the contact time period, and the temperature [45]. The assessment of the comparative impact of these parameters and the determination of the most favorable conditions is vitally important in achieving the highest adsorption. This can be achieved through various methods. The traditional approach involves batch experiments, where each parameter is tested individually while others are held constant. Alternatively, adsorption performance can be assessed at selected parameter levels, with results analyzed statistically from a limited set of experiments [46]. The response surface methodology is an effective tool for evaluating the effects of multiple factors and optimizing their levels. It accounts for interactions between factors, making it a reliable statistical approach for analyzing wastewater treatment systems. Among the available optimization strategies, the Box–Behnken design represents an efficient statistical approach derived from a three‐level fractional factorial framework, widely used for modeling and process optimization [47]. This research involved the utilization of biochar produced using the remaining mass of ground nutshell, which was functionalized with 30% tartaric acid (C_4_H_4_O_6_) to increase its capacity to adsorb the organic dye mixture of MV. In this study, methanol‐extracted groundnut shell residue was converted into 30% tartaric acid (C_4_H_4_O_6_) acid‐functionalized biochar (FGNB) and evaluated as an adsorbent for methyl violet (MV) removal. Batch adsorption experiments and Box–Behnken response surface methodology were employed to investigate adsorption behavior and optimize the operating conditions. Although biochar and activated carbon have been widely investigated for dye adsorption, the application of methanol‐extracted groundnut shell residue, an abundant secondary agro‐industrial waste, remains largely unexplored. To the best of our knowledge, this is the first study to convert this residual biomass into 30% tartaric acid (C_4_H_4_O_6_) acid‐functionalized biochar for methyl violet (MV) adsorption while integrating adsorption experiments with Box–Behnken Design (BBD) for process optimization. Various objectives were established in this investigation. To start with, biochar was synthesized with a representative agro‐waste precursor, which is groundnut (*Arachis hypogaea*) shell. Second, the biochar acquired was chemically treated at 30% tartaric acid (C_4_H_4_O_6_) to alter the physicochemical, thermomechanical, and morphological characteristics. Third, the efficiency of adsorption of the modified material was determined, and an optimization of the adsorption process was made for the removal of MV in aqueous media (synthetic type). Also, the adsorption behavior was analyzed in the framework of different kinetic and isotherm models, such as the Langmuir, the Freundlich, the Temkin, intra‐particle diffusion, and the Elovich models, to have a deeper understanding of the governing processes. Lastly, the Box–Behnken experimental design was used to optimize the process conditions and evaluate the effect of interactions among the operational variables.

## 2 Experimental

### 2.1 Materials

Groundnut samples employed in this work were sourced from a local market in Rajshahi, Bangladesh (24°22′26″N, 88°36′04″E). The chemicals were used in their acquired state and were analytical grade or better. All aqueous solutions were made with ultrapure Milli‐Q water. High‐purity methanol (CH_3_OH (99.5%) was available from Merck KGaA, Darmstadt, Germany, and all experiments were conducted using materials of consistent quality. The MV dye (solid powder) has been obtained in Germany under E Merck Ltd. The other chemicals bought in this experiment were Sodium Hydroxide (∼99%) and Hydrochloric acid (37%), which are of high purity and sourced from BDH in England. Tartaric acid (C_4_H_4_O_6_) solid crystalline powder (molecular weight around 150.09 g/mol) with a purity of 99.5% was purchased from BDH in England. Whereas the Deionized (DI) water of high purity, along with distilled water, was obtained from the Biopolymers and Sustainable Environmental Research Laboratory, Bangladesh Council of Scientific and Industrial Research (BCSIR), Rajshahi.

### 2.2 Methods

#### 2.2.1 Collection, Preparation, and MeOH Extraction of Groundnut Shell

Initially, the shells of the groundnuts harvested were selected by hand. The shells were then finely cut, cleaned well to get rid of the impurities, and they were dried under the sun (a matter of 2 h). The dry product was then milled using a mechanical mill to acquire homogeneous particles. Then the resulting coarse powder was placed in the chamber of the solvent extraction apparatus, namely, the Soxhlet instrument, for methanol extraction by following the proper protocol reported by and left to stand for around 14 h at around 60°C, whereas the solvent was methanol as well as the ratio of solid nutshell to MeOH solution was kept fixed around 1:5. Upon extraction, the extract was separated by conducting rotary evaporation meanwhile the solvents were also recovered as well as the residue was collected and was filtered through a Whatman filter paper (150 mm diameter). The filtrate was stored for later analysis, and the rest of the solid residue was also used as a precursor to the production of FGNB biochar. A schematic illustration of groundnut shell waste processing into functionalized biochar is demonstrated in Figure 1.

**FIGURE 1:**
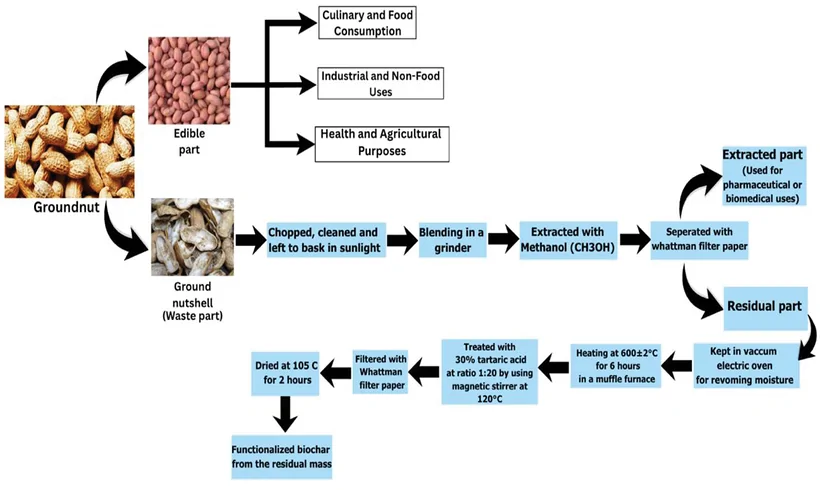
The sequence of physicochemical pretreatment, thermochemical carbonization, and acid‐mediated activation of waste ground nutshell to FGNB during the experimental procedure.

#### 2.2.2 Synthesis of Raw Biochar From the Residual Mass of Ground Nutshell

The residual mass obtained from ground nutshell powder (after phytochemicals isolation via MeOH extraction) was first subjected to drying to eliminate any remaining moisture. The material was placed in a vacuum oven (ED‐115, Binder, Germany) and maintained at 105°C ± 1°C for approximately 2 h to ensure the final removal of moisture and other residual chemicals/fractions. After drying, the material was further processed using a mechanical grinder (mixture grinder, India) to obtain a fine and homogeneous powder. Subsequently, the finely ground powder was transferred into a crucible, covered with a lid to limit oxygen availability, and subjected to thermal treatment in a muffle furnace (L‐31C6, Germany). Thermal conversion of the biomass into biochar was performed under oxygen‐limited conditions to promote controlled carbonization. The solvent was evaporated under ambient laboratory conditions. This was maintained at approximately 600°C ± 2^∘^C until the weight of the residue material in the crucible did not increase any further, a process that in turn was timed at 6 h, which was recorded using a thermosensitive pyrometer. The combustion process was very strictly controlled and controlled in an outdoor system by not allowing a direct combustion process, and an open‐air heating process guaranteed a stable carbonization process and optimized the production of biochar. Next, the resulting biochar was collected from the muffle furnace and was transformed into a fine powder through hammer milling. A schematic representation of the preparation route for FGNB is provided in Figure 2.

**FIGURE 2:**
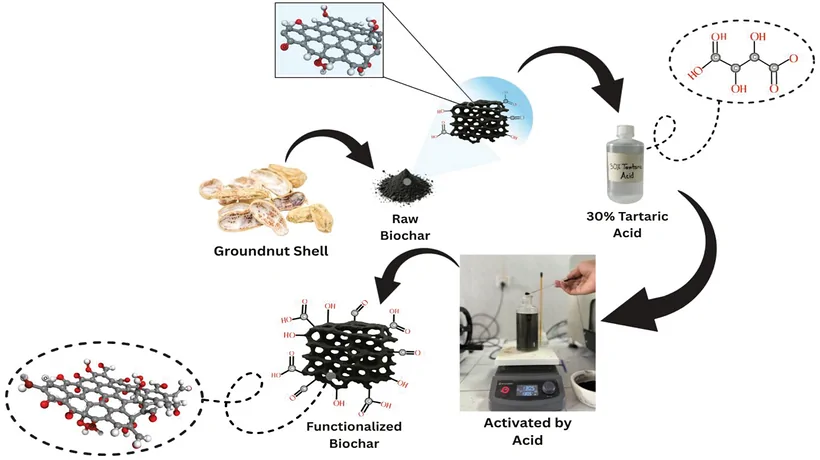
Flow diagram of the appraisal stage‐by‐stage process of preparing FGNB, which includes Soxhlet extraction of ground nutshell to the target biomass, thermochemical carbonization to produce the raw biochar, and acid‐mediated activation to generate the final high‐performance adsorbent.

#### 2.2.3 Activation of Raw Biochar by Tartaric Acid

The obtained biochar was subsequently functionalized using a 30% tartaric acid (C_4_H_4_O_6_) solution at a mass‐to‐volume ratio of 1:20 by using a magnetic stirrer at 120°C with continuous stirring at 1500 rpm for 4 h (shown in Figure 2).

Biochar prepared from ground nutshell is subjected to 30% tartaric acid (C_4_H_4_O_6_) functionalization, as it is worth noting that the incorporated tartrate (C_4_H_4_O^−^) groups, abundant with lone pair electrons, play a key role in boosting adsorption performance by forming coordination bonds between the adsorbate and adsorbents through the chemisorption procedure. After completion of the reaction, the obtained product was allowed to cool to room temperature under dark conditions. After cooling to ambient temperature, the material was separated by filtration using Whatman filter paper and rinsed thoroughly with a freshly prepared 30% tartaric acid (C_4_H_4_O_6_) solution to eliminate residual impurities. Then dried in an electric oven and preserved in a moisture‐proof screw‐cap Duran bottle for further characterization and application in crude industrial wastewater treatment for the selective separation of the targeted MV dye molecules via adsorption technology.

### 2.3 Batch Adsorption Experiments

To determine the influence of key variables of operation, such as but not limited to contact time, overall dye concentration, solution temperature, initial pH, and adsorbent dosage, on the adsorption behavior of FGNB, batch adsorption experiments were carried out. The effect of each variable was then evaluated in a systematic manner using a parametric approach. In each experimental work, 100 mL of a pre‐prepared solution of MV of a predetermined concentration with the appropriate pH was placed with a constant amount of adsorbent. The mix was rotated in an orbital shaker (FIRSTEK, Model S300R, Serial No. 230915012) at 250 rpm under isothermal conditions at 45°C. Afterwards, aliquots were drawn, filtered using Whatman filter paper to eliminate solid particles, and the clear supernatant was collected to be analyzed further. An elaborate detail of these key parameters that have an influence is in Table 1. The residual concentration of MV in the solution was quantified using a Shimadzu UV‐3600i Plus UV–vis‐NIR spectrophotometer at its maximum absorbance wavelength of 584 nm. Adsorption performance was determined by the calculation of the dye removal efficiency (R%), equilibrium adsorption capacity (R*_eff_*) using initial and final concentrations of the dye, known solution volume, and mass of the adsorbent. A systematic experimental approach was adopted to enquire into the influences of the significant operating factors, such as pH, adsorbent dosage, initial dye concentration, contact time, and temperature. The experiments were conducted in the same form in a series of experiments with only one parameter varied to ascertain the single effect of that parameter on the adsorption process. The best parameter value was then introduced in the analysis of the next variable. The percentage of dye removal (R%) and the adsorption capacity (mg/g) of the FGNB was estimated using the following equations: 1 and 2, respectively. Table 1 shows the highest percentage of dye removal (R%) and adsorption capacity (mg/g) FGNB on the effect of the various parameters. The percentage removal (R%) of the dye was calculated using Equation (1), while the adsorption efficiency at equilibrium, R_eff_ (mg/g), was computed with the help of Equation (2).

(1) $$\mathrm{Dye}\;\mathrm{removal}\;\mathrm{percentage},\;\;R\%=\frac{C_{i}-C_{0}}{C_{i}}\;$$

(2) $$\mathrm{Efficiency},\;\;R_{eff}=\frac{(C_{i}-C_{o})\times V}{M}\;\times 100$$

Where *C_i_* and *C*_0_ (mg/L) correspond to the initial and final dye concentrations in the aqueous phase, respectively. V (L) is the volume of the solution, whereas M (g) represents the amount of FGNB used.

**TABLE 1:** Experiment design to investigate the batch adsorption of MV dye onto bulky wastewater bodies through the application of FGNB: Effect of operating parameters and fixed conditions.

Trials	Temperature (°C)	Contact time (min)	Initial dye concentration (mg/L)	Dose (mg)	Solution pH	Max. removal percentage (R%)	Max adsorption capacity (mg/g)
1	25, 35, 45, 55, 65	60	40	60	5	71.94	43.792
2	45	30, 60, 90, 120, 150, 180	40	60	5	78.282	52.18
3	45	60	25, 50, 75, 100, 125, 150	60	5	51.7	61.489
4	45	60	40	30, 60, 90, 120, 150, 180	5	97.23	64.82
5	45	60	40	60	3, 4, 5, 6, 7, 8	87.5	58.33

### 2.4 Box–Behnken Design

Box–Behnken Design (BBD) is a statistical experimental design (a response surface methodology, RSM) that employs optimization of process variables and interaction effects among the responses of process variables. It is especially effective in the estimation of second‐order (quadratic) models without the need to have a complete factorial experimental design. BBD uses a mixed level of variables with middle and extreme levels, not involving experiments at extreme corners of the experiment; it is safer and more feasible in chemical and environmental processes [48]. The Box–Behnken design (BBD) is commonly utilized as an efficient statistical tool for optimizing important adsorption variables such as contact time, adsorbent dosage, solution pH, and initial dye concentration, while reducing the number of required experimental trials. This statistical approach facilitates the development of a second‐order (quadratic) predictive model that describes the relationship between independent variables and the response, such as adsorption capacity or removal efficiency. In addition, BBD enables the identification of significant factors and their interactions through analysis of variance (ANOVA), thereby providing a reliable framework for process optimization and a deeper understanding of system behavior [49]. Box–Behnken Design was used in the present work to statistically investigate and enhance the adsorptive removal of MV dye in aqueous medium using FGNB. The optimization model incorporated three main independent variables, namely, adsorbent dosage (B: 0.1–0.3 g/100 mL), initial dye concentration (C: 25–40 mg/L), fixed solution volume (100 mL), contact time (A: 30–150 min), and pH (5.0). Design‐Expert Software (Version 11.0, Stat‐Ease Inc., Minneapolis, USA) was used to analyze the data and test the importance of the linear, interaction, and quadratic effects of the variables (−1 (low), 0 (center), +1 (high)) to the adsorption process. A 100 mL of MV solution of desired concentration (25 mg/L to 40 mg/L pH 5) was transferred to a 250 mL polyethylene bottle, and 0.1 g to 0.3 g of FGNB was added to the solution, as per each run. The mixtures were then spun by an orbital shaker (250 rpm) at the desired contact time (30 min to 150 min) at 45 to adsorb the dye and afterwards, to establish the efficiency of dye removal (mg/g), the suspension was filtered, and measures of the filtrates were taken in a UV–vis spectrophotometer at a wavelength of 520 nm. The experimental data were also analyzed with the help of some nonlinear regression method whereby a second‐order polynomial model Equation (3) was adjusted on the responses (observed) to determine the influential model terms and provide the estimate of the most desirable conditions. Considering all the linear, quadratic, and interaction terms, the general quadratic model may be represented in the following way:

(3) $$Y=\beta_{c}+\sum \beta_{k}X_{k}+\sum \beta_{j}{X_{k}}^{2}+\sum \beta_{n}X_{k}X_{m}+\varepsilon$$

Where β_c_ denotes the intercept, β_k_ represents the linear effect of the factor X_k,_ β_j_ is the quadratic effect, β_n_ is the interaction between factors X_k_ and X_m_, and ε is the random error term [50].

### 2.5 Characterization Methods

#### 2.5.1 FTIR‐ATR Analysis

Fourier Transform Infrared‐Attenuated Total Reflectance (ATR‐FTIR) spectroscopy was employed to identify the surface functional groups present in the FGNB prepared from methanol (CH_3_OH)‐extracted groundnut shell residue. FTIR‐ATR is a strong surface‐sensitive method, which gives qualitative data on the chemical composition and functional moieties involved in the adsorption processes [51]. FTIR is applied to provide a definitive molecular fingerprint of surface properties of a chemical bond, such as hydroxyl (─OH), carbonyl (C═O), aromatic (C═C) features, by monitoring the infrared radiation absorption of the chemical bonds of the surface at characteristic wavenumbers. These functional groups may also provide functional sites of hydrogen bonding, electrostatic attraction, pi‐pi interactions, and complexation with dye molecules, including the MV molecule. The variation of peak positions or intensities observed before and after adsorption can provide information about the reactions of the biochar surface with the adsorbate. The positions of functional groups in the adsorption process can be clarified by these changes [52, 53]. Comparative analysis before and after dye adsorption typically shows noticeable shifts or changes in peak intensity, particularly in the ─OH, C═O, and aromatic regions, confirming the involvement of these functional groups in adsorption. These observations indicate that several interaction pathways, such as electrostatic forces, hydrogen bonding, and π‐π stacking interactions between the adsorbent surface and dye molecules, contribute to the adsorption of MV onto the biochar surface. Overall, FTIR‐ATR analysis confirms that the biochar possesses a rich array of oxygenated and aromatic functional groups, which significantly enhance its adsorption capability by facilitating strong interactions with dye molecules. Measurements of the sample at every point of the project were made using a Fourier Transform Infrared Spectrometer with Attenuated Total Reflectance (FTIR‐ATR). It was a PerkinElmer L1600300 Spectrum (Serial numbers 115, 061) of Llantrisant, UK. The samples were directly placed onto the diamond ATR crystal without the use of potassium bromide (KBr) pellet preparation. Isopropyl alcohol was used to wash the prism entirely before any measures were taken. One scan was done on each specimen at least three times; spectra were recorded at a wavenumber range of 400–4000 cm^−1^ to a resolution of approximately 5 cm^−1^as measured by the Deuterated Triglycine Sulfate (DTGS) detector [25]. For a more detailed evaluation of adsorption characteristics and structural features, the obtained spectra were processed and interpreted using PerkinElmer Spectrum IR software (version 10.6.2).

#### 2.5.2 XRD Analysis

The X‐ray diffraction (XRD) was conducted to determine the crystalline structure and composition of the prepared FGNB in terms of phase. During a normal XRD process, the sample of the FGNB is finely powdered so that it is even and is then subjected to monochromatic X‐rays. The diffracted X‐rays are measured as a dependence on the diffraction angle (2θ) and form a diffraction pattern that reflects the structure of the atoms in the material being studied. The received peaks are observed based on their positions, intensities, and widths to determine the crystalline phases and the level of structural order. The XRD pattern of FGNB usually displays a broader diffraction peak at 2θ  =  20 − 30°, which is typical of amorphous carbon structures that develop in the process of disordered placement of aromatic layers in the pyrolysis process. This amorphous form is favorable to adsorption practices in that it is linked to increased surface heterogeneity and more active sites. Besides the broad hump, sharp peaks can be observed because of the existence of crystalline mineral phases, silica or calcite that were products of the inorganic constituents of the biomass. The intensity of these peaks is usually lowered by means of chemical modification or acid treatment, which is evidence that mineral impurities have been removed and that the carbonaceous structure is improved. The diffraction behavior follows Bragg's law in Equation (4).

(4) nλ=2dsinθ

where d is the interplanar spacing, and θ is the diffraction angle. Changes in peak position and spacing provide information about structural rearrangements within the carbon matrix. The crystallite size of FGNB can be estimated using the Scherrer equation in Equation (5).

(5) $$D=\frac{K\lambda }{\beta \cos\theta }$$

In this expression, D represents the crystallite size, K is the shape factor (typically taken as ≈0.9), λ denotes the wavelength of the incident X‐ray radiation, β corresponds to the full width at half maximum (FWHM) of the diffraction peak, and θ is the Bragg diffraction angle. Smaller crystallite sizes indicate a higher degree of structural disorder, which is favorable for adsorption processes. The degree of crystallinity of FGNB can be evaluated using the crystallinity index (CrI), which is commonly calculated using the Segal method in Equation (6).

(6) $$C_{r}I=\frac{I_{002}-I_{am}}{I_{002}}\;$$

where *I*_002_ represents the utmost intensity of the crystalline peak (typically around 2θ ≈ 22°), and *I_am_* is the intensity corresponding to the amorphous region (around 2θ ≈ 18°). A lower crystallinity index indicates a more amorphous structure, which is generally associated with enhanced adsorption performance due to increased surface irregularities and active sites. In general, XRD analysis reveals that the biochar used in adsorption has an amorphous carbon structure with a small part of crystalline impurities. The structural changes that decrease crystallinity and enhance disorder are good news for adsorption because such changes render surface reactivity and accessibility of adsorption sites.

#### 2.5.3 Zeta Potential Analysis

Zeta potential analysis was conducted to evaluate the surface charge characteristics and colloidal stability of the FGNB derived from methanol (CH_3_OH)‐extracted groundnut shell residue. Zeta potential represents the electrical potential at the slipping plane of particles dispersed in a liquid medium and is a critical parameter for understanding electrostatic interactions between adsorbent surfaces and charged pollutants [54, 55]. Zeta potential, which is the electrokinetic potential located at the slipping plane of a colloidal particle, is a good indicator of the net electrical charge carried by the particle surface. Its determination is carried out routinely by electrophoretic light scattering, and the corresponding values are expressed in millivolts (mV). As a result, zeta potential serves as a key indicator for evaluating the stability of colloidal systems as well as the surface charge characteristics of adsorbent materials in aqueous media. A high absolute zeta potential value, regardless of whether it is positive or negative, generates strong electrostatic repulsive forces between particles, thereby promoting dispersion stability; conversely, when the values of zeta potential are close to zero, the system will be prone to aggregation. In adsorption studies, the zeta potential is used to define the change of surface charge with pH and functionalization, which has a direct effect on the interaction between adsorbent and adsorbate species. In adsorption studies, zeta potential is an important tool to understand the surface charge of the adsorbent. By graphically representing the net surface charge over a continuum of pH values, researchers can gain a strong predictive framework for the electrostatic companionship between the adsorbent and charged pollutants, such as the MV cation, over a range of solution conditions. The pH value at which the zeta potential disappears is called the isoelectric point, which is the exact condition in which the surface has no net charge. Consequently, there are marked transitions in adsorption efficiency close to this critical pH corresponding to a change from attractive to repulsive interfacial forces. Thus, the zeta potential data provide a consistent explanation for the observed pH‐dependent variations in the adsorption capacity and directly couple surface charge polarity with speciation and ionic state of the dye.

#### 2.5.4 Optical Microscopic Analysis

The surface morphology and structure of the FGNB made using methanol (CH_3_OH) extracted residues of the groundnut shell were examined with the help of optical microscopy. Even though optical microscopy offers a comparatively lower resolution than the electron microscopy method, it can be used as a convenient method to undertake a first‐line examination of the particle distribution, surface heterogeneity, and macro‐ and meso‐scale pore characteristics [56]. The optical micrographs show that the biochar has a highly heterogeneous and irregular morphology, which is filled with fragmented structures, uneven particle dispersibility, and discrete pore‐like vacuums. The black spots on the micrograph refer to compact carbonaceous structures, and the light spots refer to porous structures or less compact structures. Such characteristics imply that the extraction and functionalization processes of methanol drastically altered the initial lignocellulosic framework of the groundnut shell, resulting in the appearance of a rough and porous surface. Such morphological modifications are crucial for adsorption applications, as they enhance the accessibility of active sites and promote effective diffusion of adsorbate molecules into the internal pore network. The observed irregular and interconnected pore structures indicate the development of a hierarchical architecture, which facilitates both surface adsorption and intra‐particle diffusion mechanisms [57, 58]. Additionally, the removal of extractives during CH_3_OH treatment likely increased surface exposure and improved the availability of functional groups responsible for adsorption. The observations were made with a Bioimager optical microscope (Model: BMU700BD, S/N: C 24030400277, China), equipped with a high‐resolution digital imaging system. The observations were made with a Bioimager optical microscope (Model: BMU700BD, S/N: C 24030400277, China), equipped with a high‐resolution digital imaging system. It can be used in revealing visible defects, aggregates, cracks, and larger pore structures, which determine how adsorbent materials react with wastewater systems. In addition, optical microscopy is non‐destructive, quick, and easy, thus making it appropriate for the preliminary morphological examination of biomass‐derived biochar adsorbents. It gives an initial description of the shape, size distribution, roughness on the surfaces, and heterogeneity of the adsorbent particle before and after adsorption. These characteristics impinge on the accessibility of active sites and the dynamics of adsorption. The optical images can be used to show macroscopic variation in color, surface coverage, and agglomeration when adsorbates like dyes bind to adsorbent surfaces and thus assist in other quantitative analyses. Optical microscopy is used to supplement other techniques like FESEM and TEM by providing a wider view and allowing rapid screening of samples before proceeding to more detailed analysis. Overall, the optical microscopic analysis confirms that the biochar possesses a heterogeneous and porous structure, which is highly favorable for adsorption processes due to increased surface area, improved mass transfer pathways, and enhanced interaction between the adsorbent and dye molecules. In our work, with the Bio View software on a connected laptop screen, real‐time microscopic images were viewed and studied in real time. To achieve representativeness and reproducibility, 5–10 micrographs were collected for each sample, after which they were subjected to morphological analysis.

#### 2.5.5 FESEM Analysis

Field Emission Scanning Electron Microscopy (FESEM) is an advanced surface‐imaging technique that employs a finely focused, high‐energy electron beam to generate high‐resolution micrographs of material morphology and microstructural features. With the use of FESEM, the surface texture, particle morphology, pore distribution, and surface heterogeneity can be observed at much higher magnifications than traditional optical microscopy, and, therefore, this technique is essential to characterize adsorbent materials at both the micro‐ and nanoscale. FESEM analysis is a common procedure that is undertaken in the field of adsorption research to evaluate the morphological properties that dictate the performance of an adsorbent. The roughness of the surface, the network characteristics of the pores, and the presence of active sites, which are revealed by the SEM images, have a significant impact on the dynamics of interactions between the veneer of an adsorbent and the adsorbate molecules that could be dyes or heavy metal ions. Compared to FESEM micrographs of the surface before and after the adsorption technique, scientists can identify alterations in surface texture that can be attributed to the binding of pollutants, identify the effectiveness of functionalization or activation steps taken on the adsorbent, and find relationships between the observed surface features and the efficiency of the adsorption process, as well as to the mechanistic processes. FESEM, therefore, offers important information on the physical properties of adsorbent materials that form the basis of the adsorption behavior of adsorbent materials in wastewater treatment processes [59]. This method facilitated extensive visualization of the roughening of the surface, diameter of pores and voids, and decorated particles, agglomeration behavior, coarse wrinkles, cracks, and morphological homogeneity [60]. Furthermore, quantitative assessments of particles or pore size and their distribution can be determined using advanced image‐processing software such as ImageJ and Microsoft Excel.

#### 2.5.6 UV–Vis‐NIR Spectroscopic Analysis

The optical characteristics and electronic transitions of the obtained FGNB were analyzed with UV‐NIR spectroscopy, since this method offers useful information about the structure and surface of carbon‐based material. The UV–vis‐NIR spectrum usually has absorption characteristics at the ultraviolet, visible, and near‐infrared wavelengths, which have been correlated to the various electronic transitions in the material. The absorption bands that are observed in the UV wavelength (200–400 nm) are usually interrelated with π‐π* transformations of aromatic C═C bonds found within the graphitic domains of the biochar structure. These movements are evidence of the existence of conjugated systems that are formed because of the carbonization process. Also, weaker absorption characteristics at this region can be attributed to π‐π* transitions that are caused by the lone pair electrons of oxygen‐encompassing functional groups like carbonyl (C═O) and hydroxyl (─OH) groups [61]. The absorption in the visible region (400–700 nm) is generally linked to the existence of the surface functional groups, structural defects, and chromophore moieties. The functional groups introduced during chemical treatment, for example, 30% tartaric acid (C_4_H_4_O_6_) modification, can contribute to the light absorption in this range because of the higher surface heterogeneity and the appearance of other electronic states. This activity indicates the change of the electronic structure and the successful functionalization of the biochar surface. The near‐infrared (NIR) area (700–2500 nm) gives data on overtones and combination vibrations of the molecular bonds, especially OH, CH, and C═O bonds. The fact that there are absorption bands in this region shows that there are different functional groups and adsorbed species on the biochar surface. The characteristics are relevant in the study of surface interactions, particularly when the adsorption of dye molecules is being studied. The spectral profile overall indicates that the biochar has a heterogeneous structure, which consists of conjugated aromatic structures and oxygen‐containing functional groups, which are crucial to adsorption performance. The increased absorption in the visible and NIR regions on the chemical treatment can be attributed to the acceleration in the functionality of the veneer and the rise of the potential of interaction with the adsorbate molecules. These results agree with those of FESEM, XRD, and zeta potential, which prove that the surface modification has a substantial effect on the physicochemical characteristics of the biochar.

### 2.6 Investigation of the Equilibrium Mechanism of Adsorption

Kinetic analysis of adsorption and adsorption isotherm behavior are fundamental in determining the performance of biochar‐based adsorption systems because the two parameters can help in elucidating the mechanisms underlying adsorption as well as the processes that could modify the rate [62]. Biosorption kinetics is mainly regulated by mass transfer processes that take place at the solid‐liquid interface, where solute transport resistance can play a large role in defining the overall rate of adsorption. In general, there are several steps in the adsorption of dissolved contaminants on solid adsorbents [63]. Initially, the pollutant species migrate from the bulk solution toward the external boundary layer surrounding the adsorbent particles, a step commonly referred to as film diffusion. Later, the adsorbate molecules move to the external surface of the adsorbent. At the instant step, the intra‐particle diffusion occurs, in which the pollutants enter the internal pore structure of the adsorbent. In the final stage, adsorption occurs at the internal active sites of the adsorbent, where the interaction may proceed via weak physical forces or through the formation of chemical bonds, depending on the characteristics of both the adsorbent and the adsorbate. The non‐linearized equations of Langmuir, Freundlich, and Temkin isotherm models are given in Equations (7), (8), (9).

(7) $$q_{e}=\frac{q_{m}K_{L}C_{e}}{1+K_{L}C_{e}}\;$$

In this equation, *q*_te_ denotes the equilibrium adsorption capacity, *C_e_* is the equilibrium concentration of the adsorbate in solution, *q_m_* represents the theoretical maximum monolayer uptake, and *K_L_* is the Langmuir affinity constant.

(8) $$q_{e}=K_{F}\;C_{e}^{1/n}$$

In the Freundlich model, *q_e_* (mg/g) represents the amount of adsorbate retained at equilibrium, *C_e_* (mg/L) is the equilibrium concentration in the liquid phase, *K_F_* is the Freundlich adsorption constant, and n denotes the surface heterogeneity (or adsorption intensity) factor.

(9) $$q_{e}=\frac{RT}{b_{T}}\;\ln\left(A_{T}\right)C_{e}$$

For the Temkin isotherm, *q_e_* (mg/g) is the equilibrium adsorption capacity, C_e_ (mg/L) denotes the equilibrium concentration of the adsorbate in solution, T is the absolute temperature (K), and R is the universal gas constant. The parameter *b_T_* is the Temkin constant associated with the heat of adsorption (J/mol), while *A_T_* is the Temkin equilibrium binding constant (L/g).

The data on adsorption were modeled with the facilities of Langmuir, Freundlich, and Temkin isotherms, which allowed to thoroughly analyze adsorption isotherms and provided information about the adsorption mechanism. All these isotherm models are based on different assumptions made about the adsorption nature, which makes them more mechanistic. The validity of the underlying assumptions of each of the isotherms can be determined by comparing the model that appears to best fit the empirical adsorption data. These assumptions also give some understanding of the adsorption mechanism. The model equations are linearized to determine the compatibility of the isotherms, the adsorption data are replaced on the model equations, and the graphs of the relationships are plotted. The higher the determination coefficient (*R* ^2^) of 0 to 1, the more the data support the selected isotherm model. The adsorption characteristics could also be described using the generalized linear form of the Langmuir isotherm because it allows determining the important adsorption parameters. Linearized equations of Langmuir, Freundlich, and Temkin isotherm models are used in Equations (10), (11), (12), respectively.

(10) $$\frac{C_{e}}{q_{e}}=\frac{C_{e}}{A_{e}}+\frac{1}{A_{s}K_{L}}$$

(11) $$\log\;q_{e}=\log K_{F}+\frac{1}{n}\log C_{e}\;$$

(12) $$q_{e}=\gamma_{t}\;\ln A_{t}+\gamma_{t}\ln C_{e}$$

Where *K*_L_ (L/mg) is portrays the Langmuir constant affiliated with the affinity between the adsorbent and adsorbate, reflecting the interaction energy. A_s_ denotes the maximum monolayer adsorption capacities (mg/g). *q*_e_ is the amount of the adsorbate adsorbed (mg/g) at equipoise. In the Freundlich model, the parameter *K*_F_ is the Freundlich model constant, vigilant the adsorption capacity, n is the heterogeneity factor, C_e_ (mg/L) represents the equilibrium concentration of the adsorbate in solution, *q*_e_ (mg/g) represents the amount of adsorbate accumulated per unit mass of adsorbent at equilibrium. The parameter K is used to estimate the adsorption energy. In the Temkin isotherm, *A*_t_ denotes the equilibrium binding constant, while R is the universal gas constant (8.314 J/mol/K). T is the absolute system temperature (318.15K), where γ_*t*_ (J mol^−^^1^) is the Temkin constant correlated with the heat of adsorption and is expressed as the ratio RT/b.

### 2.7 Adsorption Kinetics

The kinetic characteristics and diffusion mechanisms of the adsorption process were investigated by applying various models, namely pseudo‐first order, pseudo‐second order, intra‐particle diffusion, and Elovich, to the experimental data. The application of these models enabled a deeper understanding of the underlying adsorption mechanisms and helped identify the rate‐limiting steps governing the process. Variations in contact time were examined to evaluate the progression and rate of adsorption. The pseudo‐first‐order and pseudo‐second‐order kinetic equations, presented in Equations (13) and (14), were compared with the experimental results to determine the model that best describes the adsorption behavior. The suitability of each model was assessed based on the correlation coefficient (*R* ^2^), where a higher *R* ^2^value indicates a better agreement between the experimental data and the model predictions, and thus a more accurate representation of the adsorption kinetics.

(13) $$\log\;\left(q-q_{t}\right)=\log q-\left(\frac{K_{\mathrm{ad}}t}{2.303}\right)\;$$

(14) $$\frac{t}{q_{t}}=\frac{1}{k_{2}q^{2}}+\left(\frac{1}{q}\right)t$$

In this context, t denotes the contact time, *q*_t_ represents the adsorption capacity at time t, and q corresponds to the equilibrium adsorption capacity obtained from the model. The parameters *K*_ad_ and k_2_ are the rate constants associated with the pseudo‐first‐order and pseudo‐second‐order kinetic models, respectively.

Also, the intra‐particle diffusion model was used to analyze the experimental data further on the possible rate‐controlling step of the adsorption process. This model assists in determining diffusion in the pores, whether it interprets a major purpose in the overall rate of adsorption. Moreover, the Elovich model was used to explain the demeanor of adsorption, especially in a system where chemisorption was to be performed on a heterogeneous surface. The intra‐particle diffusion model and Elovich mathematical models are given in the following Equations (15) and (16), respectively.

(15) $$q_{t}=K_{\mathrm{int}}\;\times t^{0.5}+C$$

(16) $$q_{t}=\beta \ln\left(\alpha \right)+\beta \ln\left(t\right)$$

Multilinearity is characteristic of the behavior of the intra‐particle diffusion model, which is frequently modeled by the two differing linear domains of behavior with varying slopes. If the plotted lines intersect the origin, it indicates that intra‐particle diffusion acts as the sole rate‐controlling step in the adsorption process. Nonetheless, the departure of the origin implies that even more processes, including the diffusion in the boundary layer, affect the general process of adsorption. In this model, *K*_int_ denotes the intra‐particle diffusion rate constant, whereas C reflects the influence of the boundary layer thickness. In contrast, the Elovich equation characterizes adsorption behavior on energetically non‐uniform surfaces, where the rate of uptake progressively declines as surface coverage increases. This model is particularly applicable to chemisorption processes commonly encountered in gas adsorption and wastewater treatment systems. It assumes that adsorption occurs on energetically non‐uniform sites, each possessing different activation energies. In this context, α denotes the earliest adsorption rate, β represents the desorption constant, and *q*_t_ corresponds to the amount of adsorbate accumulated at time t.

### 2.8 Adsorption Thermodynamics

Thermodynamic analysis is important in determining whether the adsorption technique is feasible and how the approach takes place. The key thermodynamic parameters, namely Gibbs free energy change (Δ*G*°), enthalpy change (Δ*H*°), and entropy change (Δ*S*°), were evaluated to assess the spontaneity and energetic characteristics of the adsorption system. The Gibbs free energy change (Δ*G*°) is used to adjudicate the spontaneity of the adsorption technique and is calculated using the following Equation (17).

(17) $${\Delta G}^{\circ }=-\mathrm{RT}\ln K_{d}$$

Here, R refers to the universal gas constant (8.314 J/mol/K), T denotes the absolute temperature in Kelvin, and K_d_ represents the distribution coefficient, defined as the ratio between the adsorbate amount per unit mass of adsorbent and its corresponding equilibrium concentration in the solution. The sign and magnitude of the Gibbs free energy change (Δ*G*°) provide insight into the feasibility of the adsorption technique. Negative values of Δ*G*° signify that the adsorption occurs spontaneously, whereas positive values suggest that the process is thermodynamically unfavorable under the given conditions. The relationship between Δ*G*°, Δ*H*°, and Δ*S*° is expressed by the fundamental thermodynamic Equation (18).

(18) $${\Delta G}^{\circ }={\Delta H}^{\circ }-{T\Delta S}^{\circ }$$

To determine Δ*H*° and Δ*S*°, the Van't Hoff equation Equation (19) is commonly applied.

(19) $$\ln K_{d}=\frac{{\Delta S}^{\circ }}{R}\;-\frac{{\Delta H}^{\circ }}{RT}$$

A linear relationship is obtained by plotting ln *K*_d_ against 1/T, where the slope corresponds to (Δ*H*°/*R*) and the intercept represents (Δ*S*°/R), allowing the determination of enthalpy and entropy changes, respectively. The enthalpy change (Δ*H*°) provides insight into the nature of the adsorption process. A positive Δ*H*° value signifies an endothermic process, where adsorption is favored at higher temperatures, whereas a negative Δ*H*° suggests an exothermic process. The magnitude of Δ*H*° provides insight into the nature of the adsorption process; values below approximately 40 kJ/mol are generally indicative of physisorption, whereas higher values suggest the involvement of chemisorption mechanisms [58]. The entropy change (Δ*S*°) indicates the extent of randomness at the solid‐solution interface during adsorption. A positive Δ*S*° value signifies the growth of randomness and disorder, usually because of the movement of water molecules and the augmented mobility of adsorbate species at the interface. Conversely, a negative Δ*S*° suggests a decrease in randomness, indicating a more ordered system after adsorption. All in all, the thermodynamic parameters give detailed information on the adsorption process, such as its spontaneity, heat exchange, and structural modification at the interface. Such parameters are necessary in the determination of the feasibility and mechanism of adsorption, as well as optimization of operational conditions in practice.

## 3 Results and discussions

### 3.1 Effect of Influential Parameters

#### 3.1.1 Effect of Temperature on MV Dye Adsorption

Figure 3a illustrates the influence of temperature on the adsorption capacity of MV dye onto the prepared FGNB. The results indicate a consistent increase in adsorption capacity as the temperature rises from 25°C to 65°C, indicating that elevated temperatures favor the adsorption technique. The given enhancement could be attributed to the fact that the higher the temperature of dye molecules, the higher the level of kinetic energy, the faster their movement, and the higher the mass transfer between the bulk phase and those adsorption sites that are available to them. Additionally, higher temperatures may contribute to the activation of previously inaccessible adsorption sites and the expansion of pore structures, thereby improving the overall adsorption performance [58]. Its optimum adsorption efficiency is found at about 55°C, indicating that the adsorbent surface and dye molecules interact best at this temperature. This has most often been linked to an endothermic adsorption reaction, in which heating enhances the interactions between the adsorbates and the adsorbent by increasing the strength of the adsorbate‐adsorbent interactions, as well as by enhancing the affinity of the FGNB surface toward the dye molecules. Nevertheless, there is a minor loss in adsorption capacity at high temperatures (e.g., 65°C, and this could be attributed to the erosion of adsorption forces or the potential erosion of surfaces at high temperature. Thermal agitation may cause the adsorbent surface to interact with the dye molecules unintentionally, causing a decrease in adsorption efficiency at very high temperatures. The identical pattern is observed in past researchers, where the adsorption capacity reaches a maximum temperature after which it decreases because of the instability of the adsorbed species [64, 65]. The overall findings demonstrate that temperature exerts a pronounced influence on the adsorption process. Any subsequent reduction or plateau observed at elevated temperatures suggests the presence of limiting factors such as weakened adsorbate–adsorbent interactions or partial desorption. This behavior underscores the necessity of identifying an optimal temperature range to ensure maximum adsorption efficiency and process stability.

**FIGURE 3:**
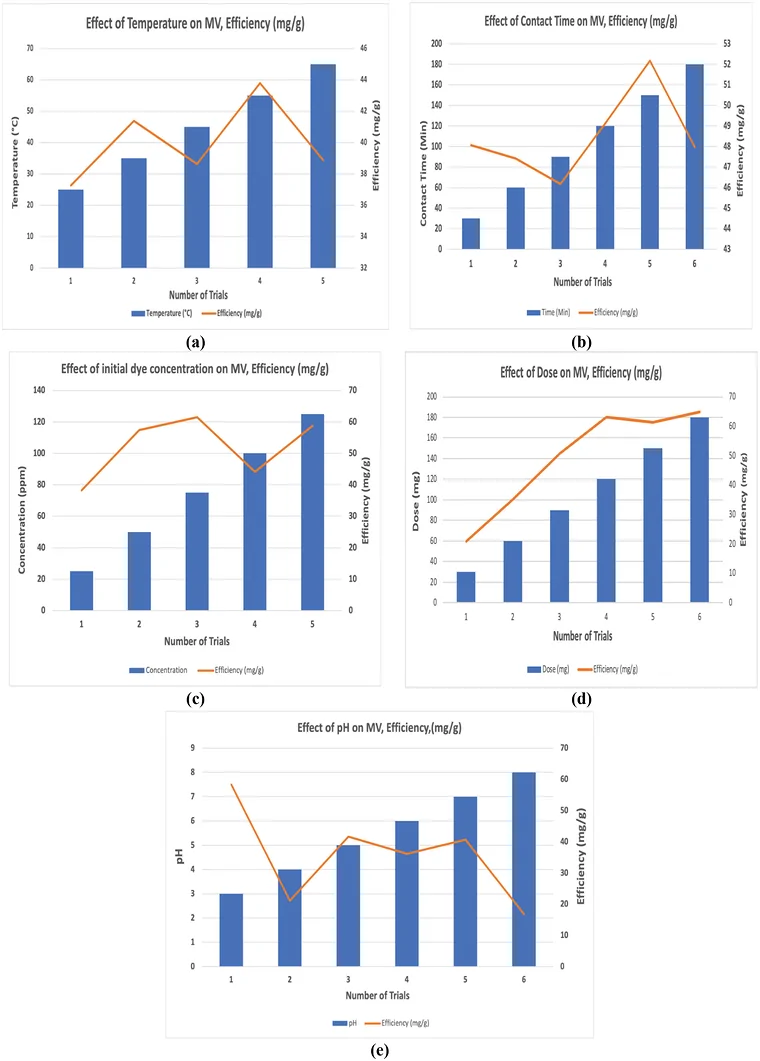
(a) The influence of temperature on adsorption of MV (adsorption condition: pH 5, dye concentration = 40 mg/L, dosage: 60 mg, contact time: 60 min, solution = 100 mL), (b) the influence of contact time on adsorption of MV (adsorption condition: pH 5, dye concentration = 40 mg/L, temperature = 45°C, dosage: 60 mg, solution = 100 mL), (c) the influence of initial dye concentration on adsorption of MV (adsorption condition: pH 5, temperature = 45°C, dosage: 60 mg, contact time: 60 min, solution = 100 mL), (d) the influence of dose on adsorption of MV (adsorption condition: pH 5, temperature = 45°C, concentration = 40 mg/L, contact time: 60 min, solution = 100 mL), (e) the influence of pH on adsorption of MV (adsorption condition: temperature = 45°C, dose = 60 mg, concentration = 40 mg/L, solution = 100 mL).

#### 3.1.2 Effect of Contact Time on MV Dye Adsorption

Figure 3b depicts how contact time influences the adsorption capacity of the MV to the FGNB, evidently indicating a highly time‐sensitive adsorption process. The capacity to adsorb is found to increase gradually with a rise in contact time, which implies that the effectiveness of uptake improves with time between the adsorbate and adsorbent. During the initial stage, the rapid uptake of dye can be attributed to the strong concentration gradient between the bulk solution and the adsorbent surface, along with the abundant availability of vacant active sites. It is the first stage, which is characterized by an increased rate of mass movement and rapid movement of dye molecules toward the exterior surface of the biochar. The richness of the accessible binding sites increases adsorption immediately; thus, adsorption capacity increases sharply at the earliest stages of the process. The adsorption rate is slowing down with the increasing contact time (60–150 min), suggesting that adsorption shifts toward surface adsorption to intra‐particle diffusion. At this podium, the dye molecules will enter further into the pores of the biochar, and the availability of active sites will start to reduce because of successive occupancy. The maximum adsorption capacity occurs at approximately 150 min, indicating that at this point, equilibrium is overshadowed. On top of this optimal contact time (e.g., 180 min), there is a slight reduction or level off in adsorption capacity. Such an action can be explained by the saturation of active locations and the formation of a dynamic balance between adsorption and desorption processes. Moreover, there is a possibility of small but significant desorption or redistribution of dye molecules in the adsorbent surface because of a longer contact period. In general, the detected trend denotes a classic adsorption process, which involves an initial fast stage and a gradual advance to the equilibrium. The findings reveal that contact time is a very important parameter that can affect adsorption efficiency and that there must be an optimum time that can result in the maximum dye removal. This is comparable to the earlier research on the dye adsorption concerning biochar and other carbon adsorbents [58, 66]. At our work, the maximum efficiency of MV by FGNB was 52.18 mg/g.

#### 3.1.3 Effect of Initial Dye Concentration

The earliest dye concentration is a critical parameter influencing adsorption performance [67]. Figure 3c reveals that the initial concentration of MV has a strong influence on the adsorption capacity of the dye. One can see a growing tendency as the concentration of the dye is increased, and when the concentration is approximately 75 mg/L, the adsorption is the maximum one can arrive at. This behavior can be explained by the enhancement of the mass transfer driving force at higher concentrations. The greater the difference in concentration between the solution phase and the adsorbent surface, the better the mass transfer, and thus, dye molecules find their way to the available active sites and efficiently occupy them. Consequently, a greater number of adsorption sites become occupied, leading to improved adsorption capacity within this concentration range [64]. At a low starting concentration, the amount of dye molecules in solution is small in comparison with the number of obtainable adsorption sites and thus optimum coverage is not achieved on the adsorbent surface. The higher the concentration, the higher the number of dye molecules that will be available to react with the active sites and thus occupy the sites, leading to increased adsorption efficiency. However, beyond the optimum concentration (around 75 mg/L), a decline in adsorption capacity is observed at intermediate levels (e.g., 100 mg/L). The reduction in adsorption performance can be explained by the progressive occupation of available binding sites, along with increased competition among dye molecules for the finite number of active sites on the biochar surface. At higher concentrations (e.g., 125 mg/L), a softened increase in adsorption capacity is again perceived, which may be due to multilayer adsorption or enhanced interactions between dye molecules and the porous structure of the biochar. This behavior suggests that while surface saturation limits adsorption at intermediate concentrations, additional mechanisms such as pore filling or aggregation of dye molecules may contribute to adsorption at higher concentrations. Overall, the observed trend highlights the significant role of initial dye concentration in governing adsorption performance. The increase in adsorption capacity at lower to moderate concentrations indicates efficient utilization of active sites, whereas the variations at higher concentrations reflect the balance between site availability and mass transfer limitations. The obtained results are in agreement with earlier reports on dye removal employing carbon‐derived adsorbent materials [58, 68].

#### 3.1.4 Effect of Adsorbent Dosage

One of the operational parameters is adsorbent dosage, which plays a significant part in ascertaining the structural properties of modified biochar and its pollutant disposal capacity. As the dosage of the adsorbent is increased, the removal is expected to improve because of increased surface area and active binding sites. Saturation will, however, not have a proportional increase in the removal efficiency when the adsorbent is added beyond an optimal dosage. Such plateau behavior is explained by the surplus of adsorption sites compared to the quantity of the pollution, combined with the rise in surface area, resulting in underutilization of active centers [63]. The reason why Ho et al. were able to arrive at the above conclusion is that they thought the greater the amount of biochar that was used, the greater the attainability of the active sites [61]. As the adsorbent dosage increases, the adsorption capacity initially shows a corresponding enhancement; however, this trend eventually approaches a plateau as the available active sites become saturated. Beyond this point, additional adsorbent does not significantly contribute to further adsorption, indicating that the system has reached its maximum adsorption capacity. However, on the other the rate of removal goes down. A suitable amount of adsorbent dose must be chosen to economize on costs, particularly in large‐scale applications [69]. The consequence of the adsorbent dosage on the adsorption performance of MV dye is depicted in Figure 3d. Per the results, there is an evident increase in adsorption capacity within the adsorbent dosage range, 30 to 120 mg. Observing the results, a clear increment in adsorption capacity can be traced; however, there is a tendency toward stabilization at high. This rise in the efficiency of the adsorption process at the beginning may be validated by the increased number of obtainable surface‐acting sites and the rise in the total surface area that is available to bind the dye. An increase in adsorbent dosage provides a greater number of available binding sites, thereby enhancing the interaction between dye molecules and the adsorbent surface, and thus, the better the required interaction occurs. At the mid‐level doses, the adsorption capacity attains close optimum values, which indicates that binding sites have been utilized. Nevertheless, above a certain dose level (over 120 mg), the rate of change decreases, which implies approaching the state of equilibrium at a slow pace. This process can relate to overlapping or aggregation of the adsorbent particles with increased concentrations that could decrease the effective surface area and interrupt access to some active sites. Moreover, constant cation of the dye in solution causes a decreased ratio of adsorbate to adsorbent at larger dosages, which causes overuse of the adsorption sites. Diffusion implications and alterations in the adsorption equilibrium dynamics are also likely to influence the minimal fluctuations that are noticed at the high doses. With the system being in the saturation stage, there is no proportional increase in the adsorption capacity according to adsorbent mass, and this underscores the need to optimize the dosage to operate the system effectively. The same trends were observed in past reports when adsorption efficiency showed an increment with adsorbent dosage to an optimum level, beyond which no further substantial increment was noticed [64, 70]. In general, the findings prove that adsorbent dosage is a significant parameter with consideration of the disposal of MV dye, and an optimum dosage is needed to achieve maximum adsorption performance with minimum use of adsorbent material.

#### 3.1.5 Effect of Solution pH

The graph exposing the effect of solution pH on adsorption is demonstrated in Figure 3e. An important parameter that has an impact on adsorption is the pH level, as it influences the electric character of adsorbents and the target ions [71]. In practical wastewater systems, the presence of various coexisting ions can significantly influence the adsorption process. These background ions often compete with target pollutants for the attainable active sites on the adsorbent surface, thereby diminishing the overall adsorption efficiency of biochar‐based composites [72, 73]. Such competitive interactions can alter adsorption behavior, particularly under conditions of elevated ionic strength. For instance, Li et al. observed that the removal efficiency of fluoroquinolones (FQs) using magnetic manganese dioxide‐modified biochar declined as the ionic strength of the solution increased. This behavior was explained by the influence of chloride ions associated with FQs, along with the presence of competing cations in the solution, which interfered with the interaction between the adsorbate molecules and the adsorbent surface [74]. The dominance of solution pH on the adsorption behavior of MV dye is illustrated in Figure 4e. The results clearly demonstrate that pH exerts a substantial influence on the adsorption techniques, with noticeable variations in adsorption capacity observed across the investigated pH range. It has a higher adsorption efficiency at a relatively elevated pH (around 3), probably because in acidic conditions, the electrostatic interactions that occur between the positively charged dye molecules and the negatively charged functional groups on the biochar surface become favorable. When the pH level is lower, the surface of the adsorbent might be protonated, but certain interactions like π‐π stacking and van der Waals force can also make successful adsorption. With a rise in pH closer to neutral (pH 6), the adsorption capacity is moderate. This behavior can be clarified by the reduced intermolecular attraction among dye molecules, along with intensified competitive interactions at the adsorbent surface, particularly in the presence of hydroxyl ions in the solution. The surface charge of the biochar starts to change in this pH range, which affects the availability and activity of adsorption sites. Beyond higher pH values (pH 7–8), the adsorption efficiency is observed to decrease significantly. The reason behind this reduction is that the concentration of OH ‐ions in solution when adsorbing the dye increases; these ions may occupy the adsorption sites of the dye and cause electrostatic repulsion in the case of negative surface charges. Also, the alterations in the dye speciation and the weakening of the adsorbing forces between the adsorbate and adsorbent may further restrict adsorption at an alkaline condition. This general pattern shows that an acidic environment prefers the adsorption of MV dye, and therefore, pH must be optimized to get the best removal efficiency. This trend has been in line with already existing literature on cationic dye adsorption involving carbon‐based adsorbents, in which electrostatic forces and surface functional groups have predominantly contributed to the adsorption behavior [58, 68].

**FIGURE 4:**
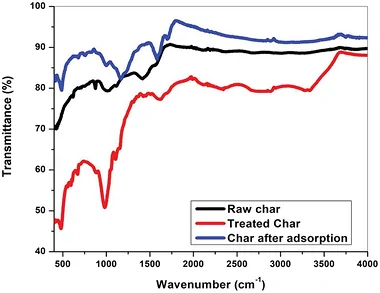
Comparative FTIR‐ATR spectra in the range of 500–4000 cm^−1^ illustrating surface functional group variations of ground nutshell biochar, 30% tartaric acid (C_4_H_4_O_6_)‐treated FGNB, and treated FGNB after MV dye adsorption.

### 3.2 Findings Based on the Characterization Methods

#### 3.2.1 FTIR Analysis

The FTIR spectra of raw biochar, treated biochar, and biochar after MV adsorption are shown in Figure 4. FTIR analysis gives a good understanding of the surface functional groups, which are also major in the adsorption process. The wide absorption band at 3200–3600 cm^−1^ is related to the stretching vibration of hydroxyl (─OH) groups, being attributed to cellulose, hemicellulose, lignin residues, and adsorbed water on the biochar surface. The bands obtained around 2920–2850 cm^−1^ are due to aliphatic C─H stretching vibrations and signify the existence of residual organic carbon structures [75, 76]. A notable band in the 1600–1700 cm^−1^ zone is related to C═O stretching of carboxyl and carbonyl groups as well as aromatic C═C vibrations, a reflection of the partially aromatic nature of the biochar matrix. There are peaks in the range of 1200–1000 cm^−1^resulted from the C─O stretching vibration of alcohol, phenolic groups, and ether groups. Absorption peaks observed near 2920 cm^−1^ and 2850 cm^−1^correspond to the asymmetric and symmetric stretching vibrations of aliphatic C─H bonds. These features are present in all samples; however, a slight reduction in their intensity after adsorption suggests possible coverage of the surface by dye molecules [77]. Following the chemical treatment, the treated char shows an increased band intensity and minor peak shifts, especially in the O─H, the C═O, and the C─O regions, indicating successful surface functionalization. The enhancement of this band compared to the raw biochar confirms successful functionalization. It can be informed in these spectral changes of the inclusion or exposure of more oxygen‐carrying functional groups to elevate surface polarity and the presence of active adsorption sites. The post‐adsorption FTIR spectrum of the char indicates that there is an evident peak shifting, attenuation, and broadening, particularly in the O─H (∼3400 cm^−1^) and C═O/C═C (∼1600 cm^−1^) regions. The observed spectral shifts confirm the occurrence of strong interactions between MV molecules and the surface functional groups of the biochar. The adsorption process is mainly driven by electrostatic forces between positively charged dye species and negatively charged surface functionalities, along with hydrogen bonding and π‐π interactions occurring between the aromatic rings of MV and the graphitic structures of the biochar. The noticeable alteration in functional group signatures following adsorption clearly indicates the significant contribution of surface chemistry to the selective adsorption of MV. In addition to aromatic C─H out‐of‐plane bending modes and carbonate (CO_3_^2−^) bending vibrations (≈710–750 cm^−^^1^), which may be related to CaCO_3_ or *K*_2_CO_3_ [78, 79]. Contributions near 800–1000 cm^−^^1^ may result from Si─O─Si symmetric stretching or overlapping C─O vibrations.

#### 3.2.2 XRD Analysis

XRD patterns of the raw char and chemically treated char are demonstrated in Figure 5 to examine changes in crystallinity and structural ordering after surface modification. Both samples exhibit a broad diffraction hump in the range of 2θ ≈ 20–30°, which is characteristic of amorphous carbon structures typically observed in biomass‐derived biochar. This broad peak corresponds to the plane of turbo static carbon, indicating a disordered arrangement of aromatic carbon layers. In the raw char, several weak and irregular diffraction peaks are observed, suggesting the presence of residual inorganic mineral phases such as silica, calcium compounds, or metal oxides inherited from the groundnut shell precursor [37]. These crystalline impurities contribute to structural heterogeneity and may partially block active adsorption sites. Following 30% tartaric acid (C_4_H_4_O_6_) treatment, noticeable changes in the diffraction pattern are observed. The treated biochar shows a reduction in the intensity and number of sharp crystalline peaks, indicating the partial removal of mineral impurities through acid leaching. The overall pattern becomes relatively smoother, with the amorphous carbon hump becoming more prominent, suggesting an increase in the relative proportion of disordered carbon structures. This structural disorder is beneficial for adsorption, as amorphous carbon provides a higher density of surface defects and functional sites. A minor sharp peak observed around 2θ ≈ 60° in the treated char may be attributed to trace crystalline residues introduced or stabilized during chemical activation; however, its low intensity suggests that the overall structure remains predominantly amorphous. Also, the crystallinity index of the FGNB was found to be 64.35744%, calculated by Origin2019b software. Overall, the XRD results confirm that chemical treatment enhances the amorphous nature and structural disorder of the biochar, which is advantageous for adsorption processes. The increased disorder facilitates greater accessibility of active sites and supports improved interaction with MV dye molecules through surface adsorption mechanisms. Overall, the XRD results confirm that 30% tartaric acid (C_4_H_4_O_6_) treatment effectively modifies the structural composition of the biochar, complementing the morphological changes observed in FESEM analysis and contributing to improved functional performance.

**FIGURE 5:**
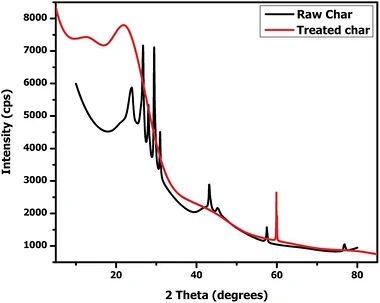
XRD of raw FGNB showing the presence of crystalline mineral phases along with amorphous carbon and 30% tartaric acid (C_4_H_4_O_6_) ‐treated biochar exhibiting reduced crystallinity and enhanced amorphous structure due to acid‐induced demineralization.

#### 3.2.3 Zeta Potential Analysis

The zeta potential of the prepared biochar was also measured, and the results are illustrated in Figure 6. The zeta potential values were found to have a range of about −75 to −5 mV with a sharp peak at −40 mV, which shows that the surface charge of the biochar is predominantly negative. The rather small and symmetric distribution indicates that the surface charge distribution is uniform, indicating the homogeneity of surface functionalization through the modification process. The negative zeta potential values can be attributed to the presence of oxygenated functional groups on the biochar surface, such as carboxyl (─COOH), hydroxyl (─OH), and carbonyl (C═O) moieties. Such groups are more likely to deprotonate in aqueous conditions, resulting in the creation of negatively charged sites (─COO), which add to the total surface charge [80, 81, 82]. The maximum at a value of about −40 mV demonstrates that the surface charge density is large and therefore, functional groups can be satisfactorily ionized, and surface modified. By conventional standards, zeta potential values exceeding or below ±30 mV (in magnitude) represent good colloidal stability, and therefore, the biochar particles are well‐dispersed in solution because of high repulsive electrostatic forces [54]. The magnitude of the negative surface charge plays a crucial role in determining adsorption performance, particularly for cationic contaminants such as MV. Enhanced adsorption occurs due to the electrostatic attraction between the negatively charged biochar surface and the positively charged dye species, and supplements other adsorption mechanisms, including π‐ π interactions and pore filling. The wide distribution to the negative side (up to −70 mV) also suggests the existence of the active sites with high binding ability, which could lead to a better adsorption activity. Also, the similarity of zeta potential distribution indicates that the modification process has produced homogenous functional group incorporation on the entire surface and reduced the heterogeneity of adsorption sites. This is beneficial in predictable adsorption behavior and reproducibility in practice. Overall, the analysis of zeta potential indicates that the biochar has good surface charges, which play a critical role in its efficacy as a cationic contaminant adsorbent.

**FIGURE 6:**
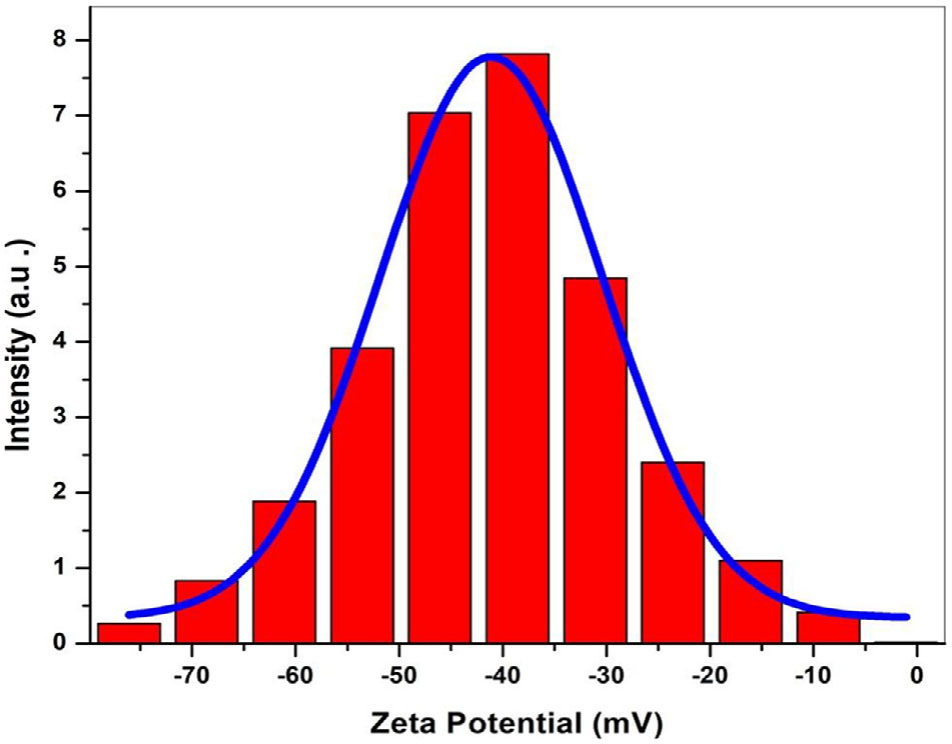
Zeta potential distribution of 30% tartaric acid (C_4_H_4_O_6_) ‐treated nutshell biochar, showing a predominantly negative surface charge with a peak around −40 mV, indicating enhanced surface functionalization and good colloidal stability.

#### 3.2.4 FESEM Analysis

The surface topography and pore structure of the biochar obtained with the help of nutshell were researched using Field Emission Scanning Electron Microscopy (FESEM) prior to and following the modification by tartaric acid (C_4_H_4_O_6_). Alongside, the pore size distribution was determined using FESEM micrographs of histogram evaluation (Figure 7). In Figure 7a, in raw biochar, it appears to be relatively dense, with several blocked or ill‐defined pores and attached particles and dust. These characteristics are likely derived from the partial degradation of lignocellulosic material and preservation of inorganic ash during the pyrolysis process, which leads to clogged pores and decreased surface area. Additionally, the degeneration of the pore edges may be caused by applying an excessive temperature for a prolonged period (600°C for 6 h) during the carbonization process [83]. After tartaric acid (C_4_H_4_O_6_) acid treatment, the surface structure shows a remarkable change (Figure 7b). The biochar surface appears more porous, rough, and broken, with well‐developed cavities and pore openings. The surface is cleaner, suggesting the successful removal of contaminants and weakly bound inorganic materials during acid treatment. This change increases the accessibility of internal pore structures and pore connectivity, thus creating more accessible active sites for adsorption. Such observations are also supported by the pore size distribution shown in the histogram (Figure 7c). The pore size distribution histogram further confirms these structural changes. The distribution is concentrated around small pore diameters, and accordingly, the pore structures are comprised predominantly of micro‐ and mesoporous. A highly skewed distribution around smaller diameters with a higher frequency in the 0 to 0.25 µm region and the highest frequency in the 0 to 0.15 µm region, and only a negligible fraction above 0.5 µm, indicating controlled pore development without structural collapse. The large number of pores in the low‐diameter range indicates a high specific surface area, and the frequency with which pore size rises increases more slowly, implying a hierarchical pore structure. This is an ideal pore structure for adsorption, with smaller pores providing greater surface area and adsorption capacity, and larger pores facilitating mass transfer and diffusion of adsorbate molecules [84]. In summary, the results of the FESEM and pore size distribution studies indicate that the use of tartaric acid (C_4_H_4_O_6_) successfully alters the structure and texture of the FGNB. The improvement in pore formation and pore connectivity and the removal of impurities is likely to refine the adsorption capacity of the biochar by providing more accessible active sites and facilitating mass transport within the biochar structure.

**FIGURE 7:**
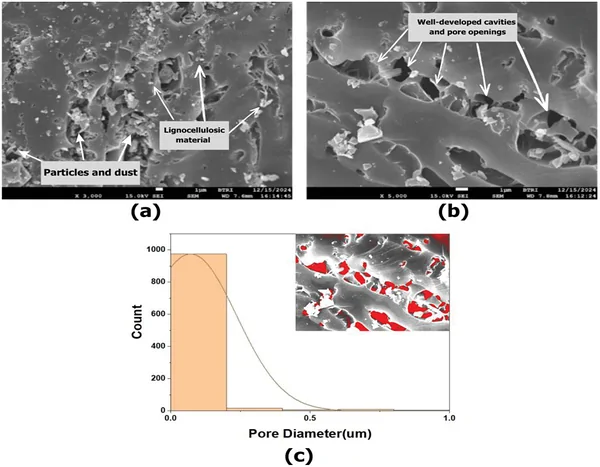
FESEM images of nutshell‐derived biochar: (a) raw biochar showing a dense and irregular surface with limited porosity, and (b) tartaric acid (C_4_H_4_O_6_) ‐treated biochar exhibiting enhanced pore development and surface cleaning, and (c) corresponding pore size distribution histogram indicating the predominance of smaller pores and the development of a hierarchical porous structure.

#### 3.2.5 Optical Microscopic Analysis

The pore distribution and surface heterogeneity of the biochar were also deliberated by optical microscopy, and the corresponding pore size distribution was evaluated through a histogram analysis, as shown in Figure 8. The structure appears non‐uniform, with a combination of fine particles and larger agglomerates distributed across the surface. The voids suggest the formation of pores during the carbonization, and the irregularity in size and shape suggests that the structure is not completely uniform [57]. The scale bar suggests that most of the features are in the micrometer range, indicating the presence of micro‐sized pores and a heterogeneous structure. The histogram and distribution curve, carried out using ImageJ and Origin Lab software, are depicted in Figure 8b and are a quantitative measure of the pore/particle size distribution of FGNB. The distribution will mostly be skewed to the smaller diameter ranges, and the maximum frequency will be recorded in lower size ranges (around 0.1–0.3 µm). This shows that there are more fine pores and microstructural features. The resulting distribution curve presents a predominance in the lower micrometer range, with most of its particles having an average diameter of less than 1 µm. The distribution curve shows a positively skewed distribution, in which the small sizes are common and where the distribution gradually drops to higher diameters, the maximum of which is about 15 µm to 20 µm. The frequency distribution curve also shows a smooth distribution curve, which further supports a unimodal distribution that is skewed to the right, with the frequency declining as the size increases. Having a few larger pores indicates a probable hierarchical construction with some smaller pores to increase surface area and larger ones to move mass. The inset binary image (highlighted with red) also indicates the segmentation regions of the pore structure to be used in quantification, image‐based, which validates the spatial distribution and connectivity of the pores in the bipolar matrix. This qualitative and quantitative analysis combined shows that FGNB has a highly developed porous structure, which is beneficial in adsorption reactions by improving the accessibility of the surface as well as diffusion routes.

**FIGURE 8:**
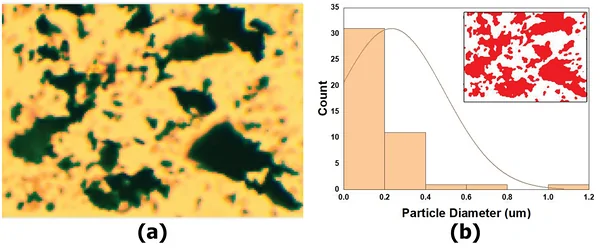
A schematic demonstration of (a) comprehensive optical microscopy‐based morphological evaluation and (b) histogram analysis of pore size distribution in FGNB.

### 3.3 Mathematical Modeling

Table 2 summarizes the calculated isotherm parameters along with the corresponding coefficient of determination (*R* ^2^) obtained from fitting the experimental data to the Langmuir, the Freundlich, and the Temkin models. Figure 9a shows the graphical models of these. These findings suggest that the three models are sufficient to explain the behavior of adsorption as shown by their reasonable comparability with the experimental results. The Langmuir isotherm is one of the applied systems that gives a very good fit, implying that adsorption is mainly made on a smooth surface by coverage of adsorbate molecules in a monolayer. The implication of this observation is the existence of uniform active sites, which facilitates the superiority of selected interactions between the adsorbent and adsorbate. Moreover, the application of the Langmuir model implies that the adsorption process can be chemisorption‐based [85, 86]. The suitability of the Freundlich model indicates that adsorption takes place on a non‐uniform surface, involving the development of multilayer coverage. This type of behavior is typically characteristic of physisorption, where interactions between the adsorbate and adsorbent are governed by relatively weak intermolecular forces [87]. The simultaneous fitting of both Langmuir and the Freundlich models indicates that the adsorption mechanism is not governed by a single process but preferably involves a fusion of physical and chemical interactions. Furthermore, the *q*_max_ values obtained from the Langmuir model indicate the maximum adsorption capacity associated with monolayer coverage of the adsorbent surface, thereby reflecting the theoretical adsorption potential of the material. Figure 9a shows the results of the adsorption behavior of MV onto the prepared biochar analyzed via the Langmuir, the Freundlich, and the Temkin isotherm models, and the results suggest an increment in the adsorbent dose and the subsequent increase in the experimental adsorption capacity. The adsorption parameters reported in Table 2, including the maximum monolayer adsorption capacity (*q*_max_ = 88.57 mg/g), were obtained using non‐linear regression of the Langmuir isotherm model.

**TABLE 2:** Parameters and determination coefficients of the Langmuir, the Freundlich, and the Temkin non‐linear isotherm models employed in this study.

Model name	Parameter	Obtained values
Langmuir	*R* ^2^ *q*_max_ (mg/g) *K*_L_	0.97196 88.57 0.007 ± 0.00183
Freundlich	*R* ^2^ *n* *K*_F_	0.94349 3.026 ± 1.20749 1.633 ± 0.22238
Temkin	*R* ^2^ *b*_T_(J/mol) *K*_T_	0.97254 100.91 0.066 ± 0.00934

**FIGURE 9:**
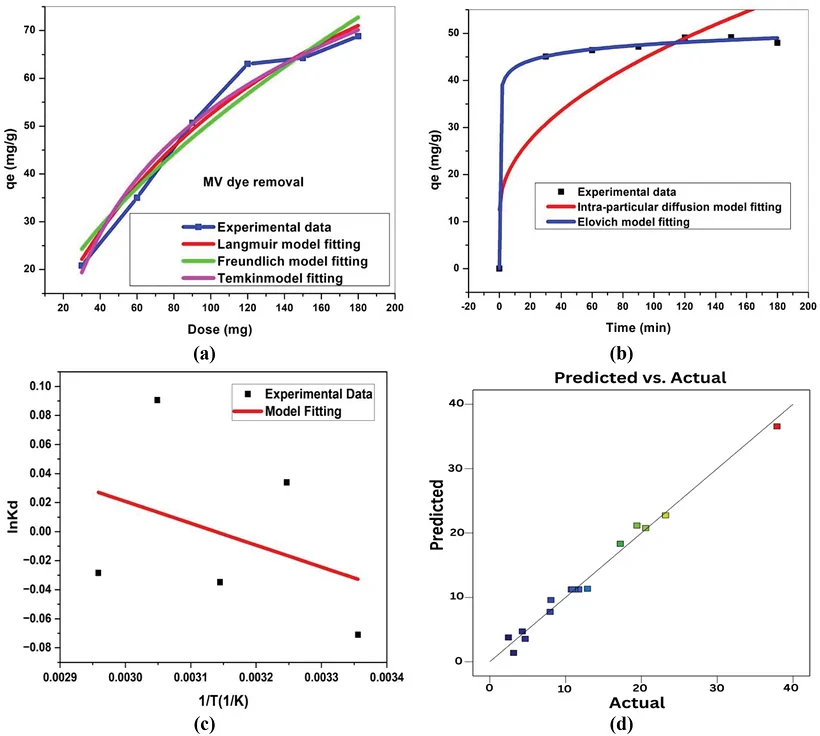
(a) Influence of adsorbent dosage on MV removal efficiency and comparative fitting of Langmuir, Freundlich, and Temkin isotherm models. The adsorption capacity (*q*_e_) increases with increasing dose, with model fits demonstrating good agreement and suggesting predominantly monolayer adsorption behavior with contributions from surface heterogeneity, (b) a comparative study based on intra‐particle diffusion and Elovich Models on the investigation of the adsorption kinetics and mechanistic behavior of MV onto tartaric acid (C_4_H_4_O_6_) ‐treated Biochar, (c) a comprehensive thermodynamic analysis of MV adsorption onto biochar: Van't Hoff Plot (lnKd vs 1/T) for determination of enthalpy and entropy changes, and (d) assessment of model accuracy and predictive performance using a parity plot of experimental versus predicted values.

The Langmuir model shows strong consistency with the experimental results, indicating its suitability for describing the adsorption system, indicating that adsorption majorly takes place in monolayer coverage on the relatively homogeneous sites. Nevertheless, the satisfactory fitting of the Freundlich model shows the existence of surface heterogeneity and non‐uniform distribution of the adsorption energies. Also, the trend in the adsorption can also be seen to be reasonably explained by the Temkin model, which means that adsorption between adsorbate and adsorbent and progressive reduction in adsorption energy in the process happen. In general, the similarity in the fit of the three models indicates that MV adsorption to the biochar is controlled by a complex of monolayer adsorption, heterogeneous adsorption to various surfaces, and variable adsorption energy, as is common in biomass‐produced adsorbents. Among the evaluated models, the Temkin isotherm exhibited the highest *R* ^2^ value, suggesting that the heat of adsorption decreases linearly with increasing surface coverage [88]. The heat of adsorption determined through the Temkin model is 100.91 J/mol. The maximum MV adsorption capacities calculated with the Langmuir model of FGNB is 88.57 mg/g and Langmuir constant *K*_L_ is 0.007 ± 0.00183. Previous investigations by Ahmad et al. and Khan et al. explored the adsorption behavior of methylene blue and Congo red dyes using biochar‐based materials. Their findings revealed that the removal efficiency of Congo red ranged from 66.8% to 96.9% for rice husk biochar and from 68.9% to 98.8% for cow dung biochar under batch experimental conditions. For rice husk‐based biochar, the Langmuir model provided the most suitable fit to the experimental data, suggesting that adsorption occurs mainly as a single‐layer coverage on a comparatively homogeneous surface. In contrast, the Freundlich model provided a better fit for cow dung‐derived biochar, suggesting adsorption on a heterogeneous surface with multilayer formation [89, 90]. According to Ahmad et al., the Langmuir equilibrium constants (K_L_) were determined to be 0.101 for rice husk biochar, 0.583 for cow dung biochar, and 0.128 for sludge‐derived biochar, indicating variations in adsorption affinity among different biochar sources. Furthermore, Aljeboree and his team demonstrated that activated carbon derived from corn cob exhibited an utmost adsorption capacity of 40.81 mg/g for MV based on the Langmuir isotherm model [91]. In a column adsorption system, Arora et al. reported that activated carbon derived from *Curcuma caesia* exhibited an adsorption capacity of 38.16 mg/g or malachite green [92]. Similarly, Foroutan et al. evaluated the adsorption performance of different activated carbon materials for MV 2B using the Langmuir isotherm model. The maximum adsorption capacities were introduced to be 26.16 mg/g for oak wood‐derived activated carbon, 37.05 mg/g for ZnO‐modified activated carbon, and 48.59 mg/g, for ZnO‐Fe_3_O_4_ modified activated carbon, respectively [93]. The adsorption capacity of malachite green on de‐ oiled soy in the study by Mittal and others was found to be 18.45 mg/g, which is also within the projections made by the Langmuir adsorption model [94]. In addition, Pooladi et al. proclaimed that the adsorption of MV onto a sawdust/Fe_3_O_4_ composite derived from wheat bran followed the Langmuir isotherm model, with a supreme adsorption capacity of 46.08 mg/g [95]. Similarly, Sevim et al. determined the maximum monolayer adsorption capacity of malachite green onto natural red clay to be 84.75 mg/g [96]. Furthermore, Tehrani–Bagha et al. proclaimed an adsorption capacity of 52 mg/g for malachite green using kaolin as the adsorbent [97].

To elucidate the kinetic stability of the adsorption of MV on FGNB, experimental data were studied with the help of pseudo‐first order and pseudo‐second‐order kinetic techniques [98]. Table 3 summarizes the results of modeling and fitting the corresponding kinetic parameters. The obtained coefficient of determination (*R* ^2^) values indicate a strong correlation between the model predictions and the experimental observations, which means that the adsorption process is determined by a set of mechanisms. This implies that the mixtures of both physisorption and chemisorption interactions play a crucial role in the entire process of adsorption. Moreover, the experimental equilibrium adsorption efficiency (R_eff_) at the end of a 150‐min contact time are 52.18 mg/g in case of MV. The similarity between the experimentally determined adsorption capacities (*q*_e_) and those estimated from the pseudo‐first order and pseudo‐second‐order models demonstrate that both approaches reliably capture the adsorption behavior of MV. This strong correlation confirms the suitability of both models in describing the kinetic characteristics of the system [87, 99]. Earlier studies by Ahmad et al. and Khan et al. confirmed that the kinetic nature of the adsorption of methylene blue and Congo red on various biochar materials is different. For rice husk, cow dung, and sludge‐derived biochar, the pseudo‐first order rate constants were reported to be 0.068, 0.018, and 0.066, respectively. On the other hand, pseudo‐second‐order rate constants were 0.031, 0.023, and 0.273 for all types of biochar used, suggesting that adsorption kinetics depend on the type of biochar [89, 90].

**TABLE 3:** Parameters and determination coefficients of kinetic and mechanism models applied in this study.

Model name	Parameters	Obtained values
Pseudo‐first order model	*R* ^2^	0.997
	*q*_e_(mg/g)	49.103 ± 0.439
	*K*	0.0813 ± 0.0082
Pseudo‐second order model	*R* ^2^	0.999
	*q*_e_(mg/g)	50.96 ± 0.307
	*K*	0.0048 ± 0.0005
Intra‐particular diffusion model	*R* ^2^	0.678
	*K*_int_	3.32 ± 0.920
	*C*	12.51
Elovich model	*R* ^2^	0.998
	α	7.83 ± 0.00036
	β	0.461 ± 0.102

### 3.4 Mechanism Model

Surface diffusion or intra‐particle diffusion usually controls the rate‐limiting mechanism in an adsorption process [98]. The experimental data were analyzed using the Weber–Morris intra‐particle diffusion model to determine the controlling step. The resultant plots missed the origin, showing that diffusion intra‐particle is not the only mechanism that controls the rate. This implies that there are other mechanisms, like diffusion in the boundary layer, which also play a role in the total adsorption kinetics. Other kinetic processes, such as boundary layer diffusion and other simultaneous rate‐limiting mechanisms, could also control the adsorption process [100, 101]. Figure 9b describes the intra‐particle diffusion and Elovich models applied to the adsorption kinetics of MV on synthesized biochar. The adsorption data show a sharply rising curve that becomes gradually level, showing the process to be sequential through many phases taking place until equilibrium. It is a multi‐step feature of an adsorption which is diffusive and by surface. The Elovich model best fits the data throughout the entire time domain, which is consistent with an energetically heterogeneous surface and indicates a significant contribution from chemisorption processes. This model also suggests that the adsorption rate decreases exponentially with surface coverage, which matches experimental observations of a rapid uptake in the initial state followed by a progressive slowdown as equilibrium is reached. Conversely, the intra‐particle diffusion model has a less accurate fit at smaller contact times, which shows that the diffusion inside the pores is not the only controlling rate process. Such a non‐linearity indicates that the adsorption process is non‐linear, meaning that there are several stages that the adsorption follows, such a process as diffusion through the boundary layer and interaction with the surface, before intra‐particle diffusion becomes influential. Thus, surface reactions and diffusion interactions determine the general adsorption process, instead of a limiting step. These results coincide with already published research on the dye adsorption of the carbon‐type adsorbents [66, 70, 102]. As indicated by the positive value of intercept (3.32 ± 0.920) for *K*_int_ obtained in this case on MV adsorption, external mass transfer and intra‐particle diffusion are part of the overall rate‐controlling mechanism [103]. The Elovich model, which was successfully employed to describe the adsorption kinetics due to the heterogeneous surface of the adsorbent [104]. The adsorption process was rapid at the initial moments (α = 7.83 ± 0.00036), then decreased gradually with the period. Similarly, Galan et al. observed similar results in the adsorption of crystal violet (CV) onto glutaraldehyde cross‐linked chitosan beads, whereby the Elovich expression fitted better than other kinetic models, thus implying a heterogeneous absorption process. At an increased surface coverage rate, fewer active sites are available for further adsorption, resulting in a dependency at later stages on the already occupied surfaces [105].

### 3.5 Adsorption Thermodynamics

The deliberated thermodynamic parameters are summarized in Table 4, while the corresponding Van't Hoff plots for MV adsorption are presented in Figure 9c. The slight increase in adsorption capacity with rising temperature indicates that the process is endothermic in nature. The positive values of enthalpy change (Δ*H*° = 472.74 J/mol), and entropy change (Δ*S*° = 4166.73 J/mol. Further support this observation, indicating heat absorption during adsorption and an increase in fortuitousness at the solid‐solution interface. The Gibbs free energy change (Δ*H*°) were found to be −1240.43307, −1282.10037, −1323.76767, −1365.43, −1407.10 J/mol at temperatures of 25°C, 35°C, 45°C, 55°C and 65°C respectively. The values of Δ*G*° were negative in the temperature range analyzed, which confirms that the adsorption is spontaneous [28]. Moreover, the progressively negative Δ*H*° values with increasing temperature indicate that higher temperatures enhance the thermodynamic feasibility of the transfer of MV onto the biochar surface. All these results suggest that the change of enthalpy (Δ*H*°), being positive, proves the adsorption process to be endothermic, where the change of entropy (Δ*S*°) is positive, hence demonstrating an increase in chaos in the adsorbent‐ adsorbate interface. Also, Gibbs free energy (Δ*G*°) values with the negative values indicate that adsorption occurs under the studied temperature conditions spontaneously [106].

**TABLE 4:** Thermodynamic parameters calculated from the experimental adsorption data of this study.

Temperature (*K*)	Intercept	Δ*S*°	Δ*H*°	Δ*G*°
298	0.47274	4.16673	1.25247	−1240.43307
308				−1282.10037
318				−1323.76767
328				−1365.43
338				−1407.1

### 3.6 Optimization Using the Box–Behnken Response Surface Methodology

Removal in terms of MV was studied by application of response surface methodology (RSM) and was done using a Box–Behnken design. First, the behavior of individual process variables was determined using parametric studies to gain an insight into their effect on adsorption behavior. The series of experiments was then carried out at the chosen levels of factors, and the data obtained were modeled and interpreted with the help of statistical modeling and the response surface. The details of the selected parameter levels are presented in Table 5, while the plan of the experiment for MV adsorption is outlined in Table 6.

**TABLE 5:** Box–Behnken factorial design levels for experimental variables.

Variable	Levels		
Time (Min)	30	90	150
Concentration of MV dye (mg/L)	10	25	40
Dose (mg)	0.1	0.2	0.3

**TABLE 6:** Evaluation of operational factors in the Box–Behnken factorial design for MV removal for optimization of MV removal percent (%) and efficiency using FGNB.

Run	Time (min)	Dose(g)	Initial Dye concentration (mg/L)	R%	Adsorption Capacity, *q*_e_ (mg/g)
1	30	0.1	25	82.42	20.56
2	30	0.3	25	51.52	4.293
3	30	0.2	10	61.11	3.148
4	30	0.2	40	86.09	17.2185
5	90	0.1	10	80.56	8.056
6	90	0.1	40	94.7	37.88
7	90	0.3	10	74.07	2.469
8	90	0.3	40	96.69	12.89
9	150	0.1	25	92.73	23.182
10	150	0.3	25	95.76	7.967
11	150	0.2	10	93.52	4.676
12	150	0.2	40	97.02	19.405
13	90	0.2	25	92.73	11.59
14	90	0.2	25	86.06	10.7575
15	90	0.2	25	93.94	11.7425
16	90	0.2	25	89.67	11.212
17	90	0.2	25	87.27	10.91

The experimental design used was based on a Box–Behnken design framework, with contact time (min), adsorbent dosage (g), and initial dye concentration (mg/L) being the independent variables, and adsorption efficiency and the equilibrium uptake, *q*_e_ (mg/g) being the response variables. The design matrix was generated using Design‐Expert 12 software. A total of 17 experimental runs, including 5 center point replicates, were conducted for MV adsorption, and the corresponding results are summarized in Table 6. Analysis of variance (ANOVA) was applied to partition the total variability of the dataset into contributions from different sources, enabling the evaluation of model significance and the testing of statistical hypotheses related to the model parameters [107]. The results, as depicted in Figure 9d, were investigated using the quadratic model proposed by the RSM tool, which is used to analyze experimental outcomes. Table 7 provides the statistical analysis of the process of MV removal.

**TABLE 7:** ANOVA for quadratic model (Response: Capacity).

Source	Sum of Squares	df	Mean Square	F‐value	p‐value	Remark
Model	1239.1	9	137.68	53.46	< 0.0001	Significant
A—Time	12.53	1	12.53	4.86	0.0632	Significant
B—Dose	481.41	1	481.41	186.93	< 0.0001	Significant
C—Conc.	595.89	1	595.89	231.38	< 0.0001	Significant
AB	0.2767	1	0.2767	0.1074	0.7527	Non‐significant
AC	0.1084	1	0.1084	0.0421	0.8433	Non‐significant
BC	94.12	1	94.12	36.55	0.0005	Significant
A^2^	2.22	1	2.22	0.8638	0.3836	Non‐significant
B^2^	51.14	1	51.14	19.86	0.003	Significant
C^2^	1.5	1	1.5	0.5815	0.4707	Non‐significant
Residual	18.03	7	2.58	—	—	—
Lack of Fit	17.31	3	5.77	32.17	0.0029	Significant
Pure Error	0.7175	4	0.1794	—	—	—
Core Total	1257.13	16	—	—	—	—

As shown in Table 7, the quadratic model was statistically significant, with an F‐value of 53.46 and *p* < 0.0001. Model terms with P‐values below 0.05 are considered statistically significant and values greater than 0.10 are considered statistically non‐significant [108]. The lack‐of‐fit test was found to be significant (*p* = 0.0029), which can be attributed to the relatively small pure error (0.7175) associated with the replicated design points — a factor known to increase the sensitivity of the lack‐of‐fit F‐test in Box–Behnken designs. However, the model exhibited a high *R* ^2^ (0.9857), adjusted *R* ^2^ (0.9672), and an acceptable predicted *R* ^2^ (0.7788), with the difference between adjusted and predicted *R* ^2^ being less than 0.2, indicating reasonable agreement. Furthermore, the Adequate Precision ratio of 28.57 (>>4) confirms a strong signal‐to‐noise ratio, supporting the model's reliability for navigating the design space and predicting adsorption performance despite the significant lack‐of‐fit. Here, the experimentally significant model terms are B, C, BC, and B^2^ in terms of MV adsorption capacity (*p* < 0.05), while A (*p* = 0.0632) was marginally significant, as its *p*‐value was less than 0.1. Among the studied variables, the initial dye concentration (factor C) exhibited the highest F‐value and the lowest *p*‐value, indicating that it has the most pronounced effect on the adsorption performance. The Box–Behnken design helped the construction of the empirical model, which relates the response variables to the governing factors via multiple regression analysis of the experimental results. In the case of MV adsorption, second‐order polynomials are used to describe this relationship, that is, the interactive effects of the independent variable, expressed in terms of equations as in Equation (20).

Capacity,

(20) $$\begin{array}{cc} q_{e} & =9.4882+0.043860\times A-140.0742\times B+1.07315\\ & \times C+0.043833\times \mathrm{AB}+0.000183\times \mathrm{AC}-3.23383\\ & \times \mathrm{BC}-0.000202\times A^{2}+348.49875\times B^{2}+0.002650\times C^{2} \end{array}$$

The optimal parameters, which are proposed to be employed in MV adsorption, according to Equation (20), are as follows: *A* = 90 min, *B* = 0.1 g, and *C* = 40 mg/L. In these circumstances, one will achieve *q*_e_ = 36. 5612 mg/g. The optimization was done by maximizing the contact time, amount, and concentration of the dye adsorbent. The calculated and experimental *q*_e_ values as per Equation (20) are plotted and illustrated in Figure 9d, and their values are presented in Table 8. To demonstrate the influences of the parameters on the response, the graphs of the response surface of the MV adsorption were drawn and presented in Figure 10. MV adsorption was plotted in three dimensions to obtain the surface plots since the process could be described using a quadratic model. The parameters in three‐dimensional representation are established in pairs along the axes, and the interactions of the two parameters on the response are illustrated simultaneously. Parameters that are not in these plots are kept fixed at their intermediate values [107].

**TABLE 8:** The predicted and actual *q*_e_ values according to Equation 20.

Time	Dose	Conc.	Actual	Predicted	Std
150	0.2	10	4.676	3.56	6
90	0.2	25	11.7425	11.34	15
150	0.3	25	7.967	7.75	4
90	0.3	40	12.89	11.34	12
30	0.1	25	20.56	20.7	1
90	0.3	10	2.469	3.78	10
150	0.2	40	19.405	21.15	8
90	0.2	25	10.91	11.34	17
30	0.3	25	4.293	4.72	3
90	0.2	25	10.7575	11.34	14
90	0.2	25	11.59	11.34	13
90	0.1	10	8.056	9.59	9
150	0.1	25	23.182	22.74	2
30	0.2	40	17.2185	18.32	7
90	0.2	25	11.212	11.34	16
90	0.1	40	37.88	36.56	11
30	0.2	10	3.148	1.39	5

**FIGURE 10:**
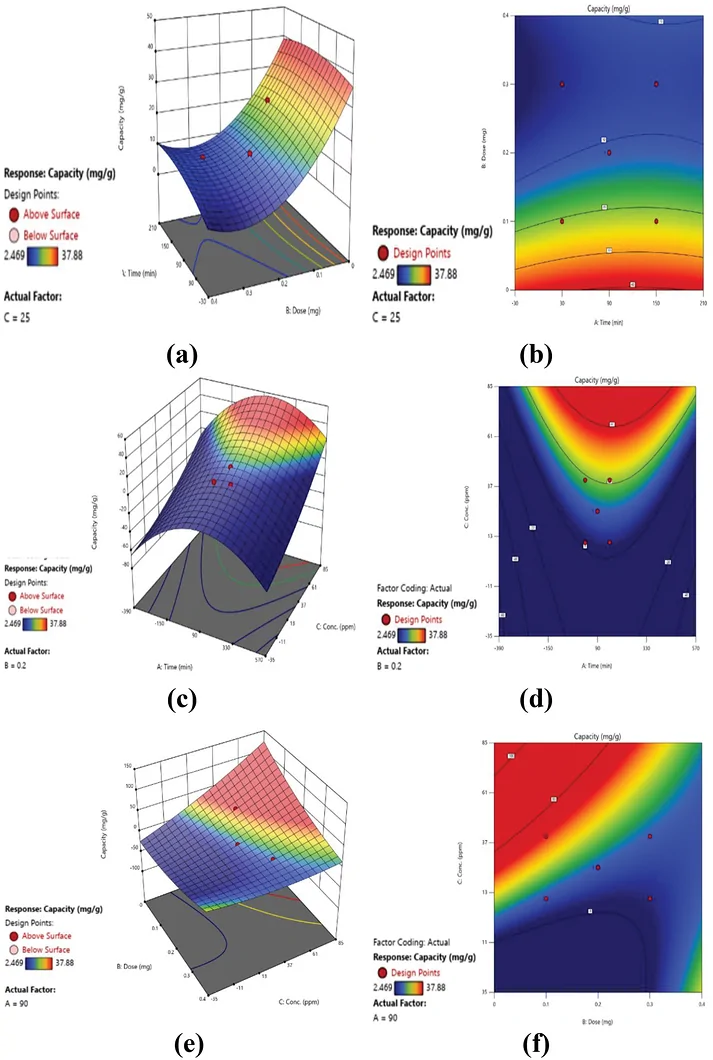
Response surface methodology (RSM)‐based optimization of adsorption capacity, showing interaction effects among key process variables via three‐dimensional surface and contour plots: (a) temperature‐dosage interaction, (b) time‐concentration interaction, and (c) dosage‐concentration interaction.

## 4 Proposed adsorption mechanism

Figure 11 illustrates a visual presentation of our suggested synergistic approach of sorption addressing the selective separation of the MV dye onto FGNB sorbents from bulky crude industrial wastewater. Activation with 30% tartaric acid (C_4_H_4_O_6_) will produce many active binding sites at the surface of the functionalized biochar. The experimental findings obtained from the FTIR, zeta potential, surface morphology, adsorption kinetics, and isotherm analyses collectively indicate that the high removal efficiency of MV by FGNB can be attributed to the combined effect of its structural and chemical characteristics. The modification process leads to the formation of a well‐developed mesoporous framework characterized by interconnected cavities and accessible pore channels, which enhance surface exposure. In addition, the treatment process enriches the surface with oxygen‐containing functional groups such as ─COOH and ─OH, thereby increasing the number of available active sites for dye binding and improving adsorption performance [109]. In addition, the aromatic carbon framework of the biochar promotes π–π interactions between the aromatic rings of methyl violet and the graphitic domains of FGNB, thereby enhancing adsorption affinity. This enhancement is primarily attributed to chemisorption, an irreversible chemical bond‐forming process (between the binding sites of sorbents and the active edge of pollutant molecules) in which the mesoporous structure of the nanosorbent facilitates intra‐particle diffusion. Electrostatic interactions also play an important role in the adsorption process [110]. Under the optimized pH conditions, the negatively charged functional groups present on the FGNB surface favor the adsorption of the cationic methyl violet molecules through electrostatic attraction. Furthermore, the porous structure of the functionalized biochar provides abundant accessible adsorption sites, enabling pore diffusion and pore‐filling mechanisms that contribute to the overall adsorption capacity. During this process, pollutant molecules migrate from regions of higher concentration in the bulk solution toward lower concentration sites within the adsorbent matrix, leading to effective adsorption. Therefore, the excellent adsorption performance of FGNB, with a maximum adsorption capacity of 88.57 mg/g, is attributed to the synergistic contribution of these complementary adsorption mechanisms rather than to any single interaction. This multi‐mechanistic adsorption behavior explains the high affinity of the prepared adsorbent toward methyl violet and highlights the effectiveness of converting methanol‐extracted groundnut shell residue into a value‐added adsorbent for sustainable wastewater treatment.

**FIGURE 11:**
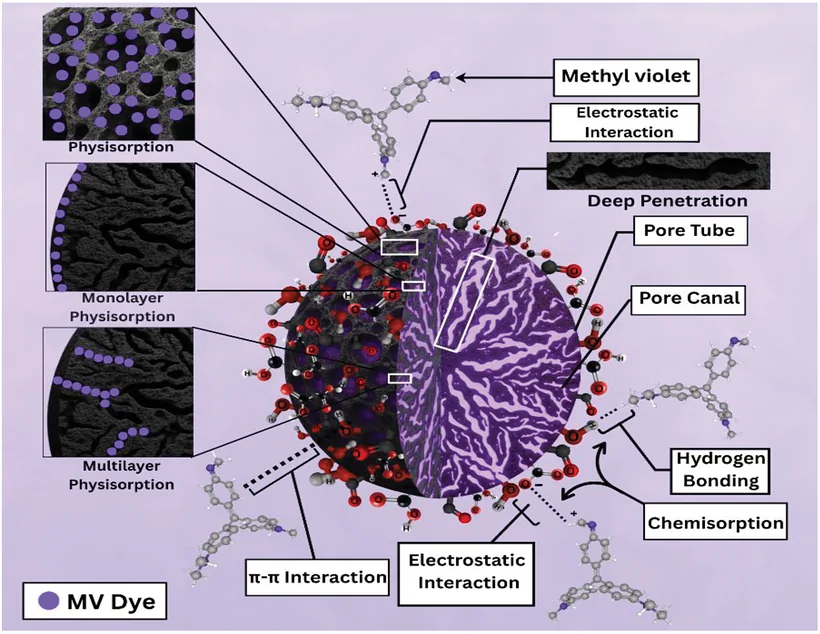
Schematic illustration of the proposed synergistic mechanism for the removal MV dye from bulky wastewater bodies by the FGNB adsorption mechanism of MV dye onto functionalized biochar, highlighting multiple interaction pathways including electrostatic attraction, π‐π interactions, hydrogen bonding, and chemisorption, along with pore diffusion processes such as deep penetration through pore channels and tubes, leading to monolayer and multilayer physisorption.

## 5 Comparative study with others

Although the impressive adsorption performance of many advanced nanomaterials and engineered composites has been reported, these materials are often expensive to produce, involve compacted preparation processes, and may raise environmental issues [111]. On the other hand, biomass‐based adsorbents like biochar have attracted much attention because of their low cost, renewability, environmentally benign nature, and surface modifiability [57, 58]. These can be derived from crop residues and further treated physically or chemically to enhance their adsorption capacity. To this end, comparisons between different adsorbents are required to assess their effectiveness, feasibility, and potential for large‐scale use. These evaluations provide important insights into the influence of characteristics such as surface area, pore structure, and surface chemistry on the adsorption performance [112]. And they help to identify sustainable solutions that offer both effective treatment and economic and environmental benefits. Accordingly, this study is accompanied by a detailed comparison with other reported adsorbents to evaluate the performance of the prepared groundnut shell biochar. This allows us to compare its adsorption performance with other materials, and it underscores its potential as an economical, environmentally sustainable, and scalable approach to wastewater treatment. Table 9 provides comparative data on the efficiency of different adsorbents applied for the disposal of toxic contaminants, like metals and dyes, under different operating conditions. It illustrates the diversity in terms of adsorption capacity, depending on the adsorbent, the pollutant, and the adsorption system being used. Various high‐tech adsorbents, including TiO_2_‐based metal composites, hybrids of graphene oxide, nanocellulose modified coal, and activated composites of chitosan, show significant removal efficiencies in the case of numerous heavy metals. For example, TiO_2_‐metal composites exhibit significant removal efficiencies for Cu (II), Ag(I), Ni (II), Cd (II), Cr (VI) and Mn (II) from photo‐deposition, suggesting their versatility. Likewise, graphene oxide/titanate composite materials display good capacities greater than 530 mg/g for Pb (II) and Cu (II), indicating the potential of carbon‐nanostructure‐based materials for heavy metal disposal. In the reported systems, some adsorbents exhibit very high adsorption capacities. Cellulose xanthate obtained from sugarcane bagasse has capacities of 712.89 mg/g for Pb (II) and 181.18 mg/g for Cu (II), while activated chitosan‐modified coal also exhibits good performance with capacities greater than 230 mg/g. Such high capacities suggest the high affinity of modified biopolymers and modified materials for metal ions, attributed to the large number of functional groups [113, 114]. By contrast, some natural and low‐cost adsorbents, like the cellulose nanocrystals (CNCs) from banana rachis and nanostructures derived from cashew nutshell, show relatively low adsorption capacities but are intriguing options due to their low cost and sustainability. Adsorption studies focused on dye removal also show high efficiencies, with advanced nano‐amide clays showing efficiencies of 99.8% for toxic dyes, suggesting their promising potential for wastewater treatment. The type of adsorption processes adopted in the studies range from batch adsorption to fixed‐bed column and continuous systems. Although batch systems are widely employed for laboratory‐scale assessment, column‐based and continuous systems are more suitable for industrial deployment because of their practicality and scalability. Crucially, this study, which uses biochar derived from groundnut shell, shows a reasonably good adsorption capacity of 64.82 mg/g for the removal of methyl dye in batch systems. While this is lower than more sophisticated materials, this aspect remains noteworthy considering the low cost, eco‐friendly nature, and straightforward preparation process of the biochar. This suggests that biomass‐derived adsorbents are promising candidates to replace more advanced and costly materials. In general, the table shows that the interaction between functional groups, pore size, and operation conditions is very significant in affecting the adsorption efficiency. While high‐tech nanomaterials perform better in terms of adsorption capacity, sustainable bio‐based adsorbents present an attractive combination of performance, cost, and environmental compatibility, making them viable alternatives for large‐scale water treatment.

**TABLE 9:** Comparative evaluation of reported adsorbents for hazardous pollutant removal: Insights into adsorption capacity, material properties, and operating conditions.

S/L	Name of hazard pollutants	Name of bionanoadsorbents	Maximum adsorption capacity	Mode of adsorption procedure	Refs.
1	Cu, Ag, Cd, Ni, Cr and Mn	TiO2‐ metal composite	46% of Cu (II), 19% of Ag (I), 16.64% of Ni (II), 14.48% of Cd (II), 5.48% of Cr (VI), 7.78% of Mn (II)	Photo deposition	[115]
2	Pb (II), Cd (II) and Cu (II)	Reduced graphene oxide@titanate hybrids (rGOTHs)	530.5, 201, and 130.5 mg/g for Pb (II), Cd (II), and Cu (II), respectively	Batch adsorption	[116]
3	Pb (II) and Cu (II)	Cellulose xanthate derived from sugarcane bagasse	712.89 and 181.18 mg/g respectively	A fixed‐bed column adsorption	[117]
4	Pb (II) and Cr (III)	Banana rachis cellulose nanocrystals (CNCs)	>10.0 mg/g and >12.4 mg/g respectively	Column adsorption	[118]
5	Ni^2+^ and Congo Red	Crystalline nanocellulose‐ modified bituminous coal (CNC‐MC)	328.70 and 478.27 mg/g respectively	Continuous column adsorption	[13]
6	Pb^2+^ and crystal violet	Activated chitosan‐ modified coal (ACMC)	243.08 and 238.51 mg/g respectively	Column adsorption	[5]
7	Toxic fuchsin dye	Advanced nano‐ ceramic clays	99.8% of dye	Continuous column flow adsorption	[119]
8	Cr and Mn (II)	Cashew nutshell (CNS)	56.40% and 53.09% respectively	Continuous adsorption	[120]
9	Acacia nilotica sawdust activated carbon (ASAC)	Indigo carmine dye (ICD)	24.67 mg/g	Fixed bed column adsorption study	[121]
10	MV dye	FGNB	88.57 mg/g	Batch adsorption	This work

## 6 Conclusion

The findings of this study show that biochar formed using groundnut shell waste material has a high prospect of being used as an efficient adsorbent in the elimination of MV in aqueous systems those that are frequently released untreated into natural water bodies. The adsorption performance was systematically assessed utilizing batch experiments, while the optimization of key process parameters was achieved through the application of Box–Behnken design within the framework of Response Surface Methodology (RSM). Also, the adsorption process was thoroughly assessed by equilibrium, kinetic, and thermodynamic parameters to have a more profound understanding of the adsorption mechanisms behind the reaction. The analysis of the obtained characterization gave the main physicochemical properties of the obtained adsorbent, and the comparative analysis of FTIR before and after dye uptake showed that there was adsorption mainly at the surface of the adsorbent. FTIR help to clarify the roles of functional groups in the adsorption process. The effect of the distinctive operational parameters was also studied methodically by carrying out batch experiments, and optimization was then done to identify optimal conditions of the technique. For an inaugural dye concentration of 40 mg/L, the optimal operating conditions were identified as natural pH, an adsorbent dosage of 60 mg, a contact time of 60 min, and a temperature of 45°C. Under these conditions, efficient adsorption performance was achieved. However, a significantly enhanced removal performance was achieved under acidic conditions, especially near pH 3, suggesting that electrostatic forces are the primary contributors to the adsorption process. Under acidic conditions, stronger electrostatic attraction between positively charged dye species and negatively charged surface functionalities enhances the adsorption efficiency. To examine equilibrium behavior, the experimental results were analyzed using Langmuir, Freundlich, and Temkin isotherm models, all of which showed high correlation coefficients. The strong agreement of both the Langmuir and the Freundlich isotherm models with the experimental data suggests that the adsorption process involves a combination of mechanisms. This indicates the simultaneous contribution of physisorption and chemisorption during the uptake of the dye onto the adsorbent veneer. The Temkin model yielded a heat of adsorption value of 100.91 J/mol, suggesting the predominance of chemisorption mechanisms. According to the Langmuir isotherm, the maximum monolayer adsorption capacity of the biochar for MV was determined to be 88.57 mg/g. Kinetic analysis further indicated that both pseudo‐first‐order and pseudo‐second‐order models adequately describe the adsorption behavior, implying that physical and chemical interactions occur simultaneously during the adsorption process. In addition, the intra‐particle diffusion and Elovich mechanism models provided further insight into the adsorption rate and underlying kinetic behavior, allowing the conclusion that both diffusion‐controlled and surface‐related adsorption take place to promote the overall kinetics. Thermodynamic evaluation indicated that the adsorption proceeds spontaneously and is thermodynamically favorable within the investigated temperature range, and the character of the heat exchange is similar, the calculated thermodynamic indicators are respected. The Box–Behnken response surface analysis furnished good quantitative data in adsorption. Accordingly, the initial dye concentration turned out to be the most important factor that affects adsorption. The 3D response surface plots revealed the effect all parameters have on the result. Moreover, polynomial equations were developed to describe the relationship between adsorption capacity and the experimental variables and can predict the outcome when the parameters are varied within the ranges that are examined, and optimization of the other adsorption conditions. These equations optimize the process conditions of the minimization of the contact time, minimization of the adsorbent dosage and concentration of dye, and the equilibrium adsorption capacity was determined under fixed operating conditions, including a contact time of 90 min, an adsorbent dosage of 0.1 g, and an initial MV concentration of 40 mg/L.

## Author Contributions

M.A.U.A.: methodology, writing – original draft, Software, investigation, validation. M.M.R: conceptualization, fund acquisition, methodology, software, validation, formal analysis, data curation, writing – review & editing, J.A.: methodology, investigation. B.C.G.: methodology, validation. S. K.: project administration, fund acquisition. *M.R.N*.: software, resources. R.A.: resources. M.I.H.: software, formal analysis, visualization, writing – review & editing.

## Funding

The authors extend their appreciation to the Deanship of Scientific Research at Northern Border University, Arar, KSA for funding this research work through the project number “NBU‐FFR‐2026‐3547‐11”. As well as to the Ministry of Science and Technology (MOST), the People's Republic of Bangladesh, and the Bangladesh Council of Scientific and Industrial Research (BCSIR), for their joint funding to conduct this current work through the Research and Development Project under grant numbers (G.O.39.02.0000.011.14.212.2026.717 and G.O.39.00.0000.207.0004.25.174).

## Conflicts of Interest

The authors declare no conflicts of interest.

## Data Availability

The data that support the findings of this study are available on request from the corresponding author. The data are not publicly available due to privacy or ethical restrictions.
